# Skin Barrier Modulation by Oleyl-Based Co-Formulants: Mechanistic Insights from Molecular Simulations

**DOI:** 10.1021/acsomega.6c00967

**Published:** 2026-08-05

**Authors:** Callum J. W. R. Ward, Conor Whitehouse, Jennifer Webb, Rebecca Notman

**Affiliations:** † Department of Chemistry, 2707 University of Warwick, Coventry CV4 7AL, U.K.; ‡ Jealott’s Hill International Research Centre, Syngenta, Bracknell RG42 6EY, U.K.; § School of Biochemistry, 1980 University of Bristol, Bristol BS8 1TD, U.K.

## Abstract

Understanding and predicting dermal absorption is important for many applications including transdermal drug delivery, personal care product development, and chemical risk assessment. Real-world dermal exposures typically involve chemically complex formulations that contain not only the active ingredient but also coformulants such as solvents, thickeners, humectants and emulsifiers, which can significantly alter skin permeability and dermal absorption. In the case of personal care and pharmaceutical formulations, penetration enhancers are often added to improve delivery of active ingredients, however the interaction of these ingredients with the skin and how they modulate skin barrier function is poorly understood. In this work, we have used molecular dynamics simulations to investigate how a series of oleyl homologues with varying polyoxyethylene (POE) headgroups, partition into model stratum corneum lipid bilayers and alter their structural organization and barrier properties. These molecules are widely used as coformulants in the chemical and pharmaceutical industries. The simulations show that all of the compounds spontaneously partition into the bilayer within hundreds of nanoseconds. Once partitioned, they disrupt the interfacial hydrogen bonding network and lipid order, reducing the free energy barrier to water permeation across the bilayer. The extent of disruption correlates with POE headgroup length, with longer POE chains causing more pronounced disruption of the interface and enhanced permeability. These simulations provide the first atomistic insights into how methyl oleate and POE oleyl ethers affect skin barrier properties. Our findings highlight the critical role of coformulants in modulating dermal absorption and provide a molecular basis to guide formulation design and support robust safety assessment of dermal exposure.

## Graphical abstract

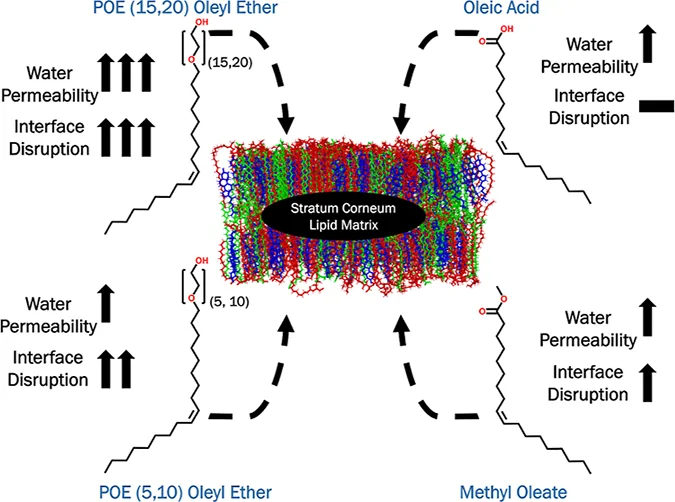

## Introduction

The dermal absorption of chemicals is of critical importance across the chemical industry. In the pharmaceutical industry, enhancing dermal absorption can be beneficial in the development of transdermal drug delivery systems.^1^ In addition, dermal absorption is a key exposure route that must be considered in the risk assessment of commercial, industrial and agrochemical formulations.^2,3^ For compounds with systemic toxicity, the dermal absorption can have a significant impact on the risk assessment and ultimate viability of the product.

For a chemical to absorb through the skin, it must first cross the stratum corneum (SC), which is the outermost layer of skin and the primary barrier to both water loss and chemical penetration.^4^ The structure of the SC is illustrated in Figure 1.^5^

**Figure 1:**
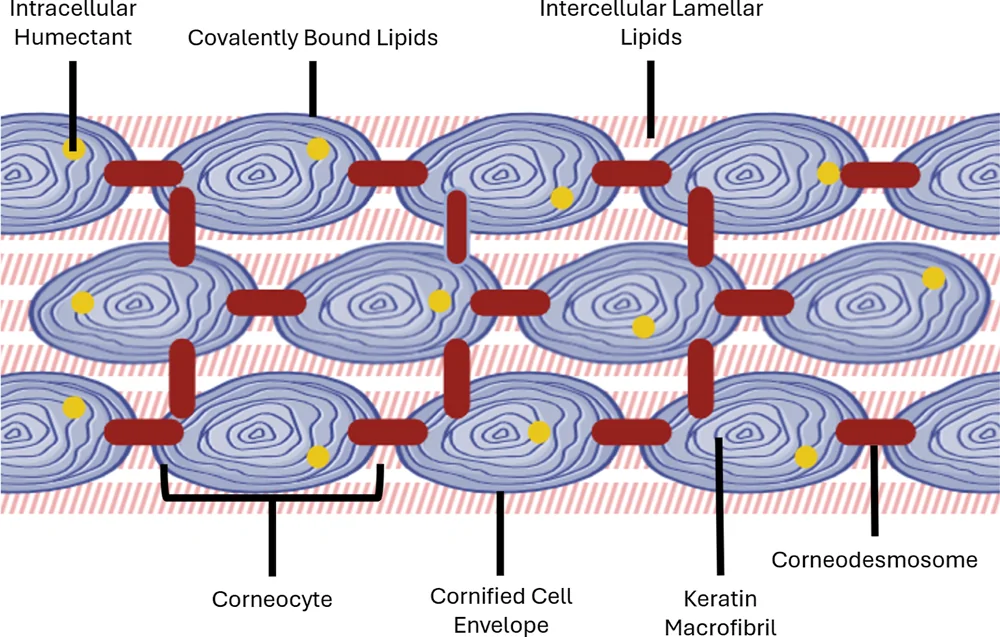
Schematic diagram of the SC skin barrier. Adapted with permission from Harding, C. R. The stratum corneum: structure and function in health and disease. *Dermatologic Therapy* **2004**, *17*, 6–15. Copyright 2004 John Wiley and Sons.

The SC has a “bricks and mortar” structure featuring flattened, rigid corneocytes embedded in a continuous multilamellar lipid matrix. This lipid matrix provides the only continuous route for chemical penetration through the SC^6^ and is chiefly responsible for the strong barrier properties of the SC.^7^ The matrix consists of a roughly equimolar mixture of ceramides, free fatty acids and cholesterol.^8^ Within this mixture there exists a broad range of ceramide and fatty acid subclasses.^9,10^ Two of the most abundant subclasses in the human SC are lignoceric acid (C24:0 saturated fatty acid, hereafter referred to as C_24_ fatty acid) and the nonhydroxy fatty acid phytosphingosine ceramide (CER­[NP]), with saturated lipid tail lengths of 24 and 18 carbons.^10,11^ The structures of the lipids used in this work are shown in Figure 2.

**Figure 2:**
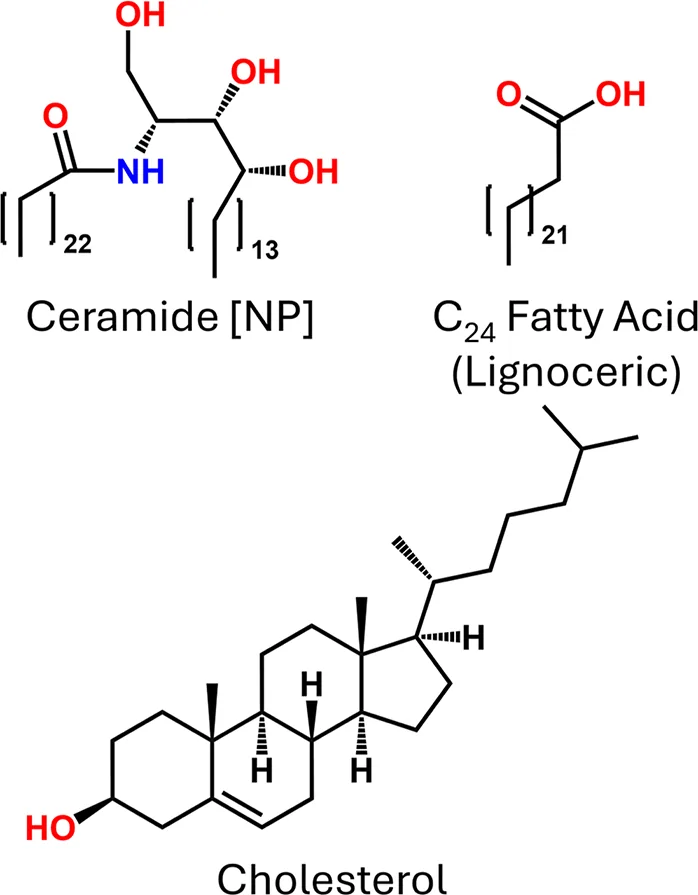
Molecular structures of CER­[NP], C_24_ fatty acid and cholesterol as modeled in this work.

In the assessment of dermal absorption, it is typical to monitor the permeation of an active ingredient of interest.^12^ However, active ingredients are rarely supplied neat, but in complex formulations designed to optimize the efficacy and safety of the product. Co-formulants within these formulations can have a substantial effect on the measured dermal absorption.^13,14^ This effect is exploited in the design of transdermal drug delivery formulations to enhance skin permeation.^15,16^

A number of common coformulants in pharmaceutical and industrial products are based on oleyl derivatives, which share a common structural motif: an 18-carbon aliphatic tail with a cis double bond on the ninth carbon. The series includes oleic acid, its methyl ester (methyl oleate) and polyoxyethylene (POE) oleyl ethers, which differ in the size of their polar headgroups. The oleyl homologues investigated in this work are shown in Figure 3.

**Figure 3:**
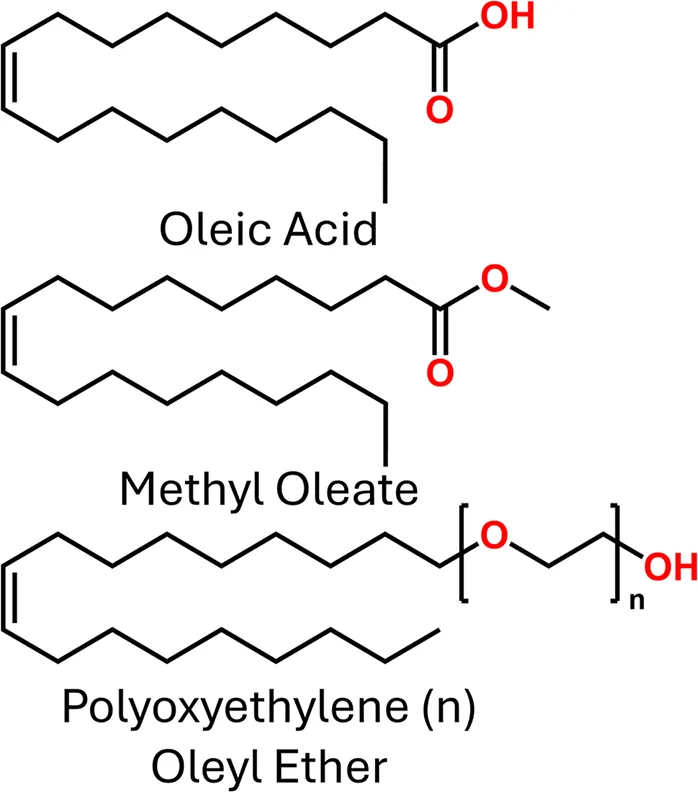
Molecular structures of the oleyl homologues investigated in this work, including oleic acid, methyl oleate and the POE oleyl ethers.

Oleic acid is widely used for its broad penetration enhancing properties. It increases the permeation of a wide range of ionic and neutral species, particularly chemicals of pharmaceutical interest.^17,18^ Experimental studies have shown that oleic acid freely partitions into and is distributed throughout the SC, where it induces disorder within the SC lipids. The exact mechanism underlying this disorder is unclear, however it is proposed to be due to lipid fluidization and, to a lesser degree, lipid extraction.^19,20^ Further experiments using SC Langmuir monolayers have shown that at lower concentrations, oleic acid preferentially associates with ceramide-enriched domains, and to a lesser extent, with fatty acid-enriched domains, inducing localized disorder within these domains.^21^

Due to its widespread use and uncertainty surrounding its mechanism of action, the effects of oleic acid on the SC have also been extensively studied by molecular simulations. Initial coarse-grained simulations of oleic acid’s interactions with dipalmitoylphosphatidylcholine bilayers showed that oleic acid was able to freely and uniformly partition into lipid bilayers, resulting in a minor increase in water permeability and a decrease in the bilayer area compressibility modulus.^22^ United-atom molecular dynamics (MD) simulations of model SC lipid bilayers comprising nonhydroxy sphingosine ceramide (CER­[NS]), C_24_ fatty acid and cholesterol found that low concentrations of oleic acid (0–0.1 mol %) only caused minor decreases in both the bilayer thickness and density.^23^ More recent coarse-grained simulations on multilayer SC systems consisting of equimolar CER­[NS], C_24_ fatty acid and cholesterol, have shown that oleic acid freely partitions into the lipid layers and causes a slight decrease in lipid order.^24^

Methyl oleate is a common coformulant used across a range of industries. It is known to act as a dermal penetration enhancer. For example, its addition to a propylene glycol vehicle has been shown to enhance the uptake of chlorpheniramine maleate by ∼70%.^25^ Additionally, methyl oleate has been investigated as a penetration enhancer for buccal epithelium administration (through the inner lining of the cheek), and was found to increase the uptake of propranolol through a suggested mechanism of fluidization of the intercellular lipids.^26^ Although its exact mechanism of action in the skin remains unclear, topically applied methyl oleate has been shown to penetrate into the deeper layers of the epidermis and disrupt lipid metabolism, suggesting that it is capable of freely partitioning through the skin barrier.^27^

Finally, we consider the POE oleyl ethers, which feature a variable length POE headgroup, denoted hereafter POE­(*n*), where *n* is the number of ethylene oxide units. These molecules are widely used as surfactant coformulants. Their properties can be tuned by varying the length of the oxyethylene chain.^28^ Experimental studies have revealed that POE oleyl ethers can significantly disrupt the skin barrier, reducing the lipid content, decreasing the skin thickness and lateral packing density and increasing SC fluidity.^29^ Longer chain POE oleyl ethers i.e. POE(20) had the greatest disruptive effect. Further work showed that POE fatty acid cetyl and stearyl ethers significantly increased the rate of transepidermal water loss, with POE(20) again producing the strongest effect. Interestingly, this effect was greatly reduced for ethers with longer POE chains than 20 units, where the observed transepidermal water loss was decreased for POE­(40 and 100) stearyl ethers.^30^

Despite numerous experimental and simulation studies investigating the penetration enhancing properties of common oleyl-based coformulants, the molecular mechanisms that govern their effects on the skin barrier remain poorly understood. In this study, we have performed MD simulations to explore the interactions between a SC lipid bilayer model and a homologous series of oleyl-based compounds: oleic acid, methyl oleate and POE oleyl ethers with 5, 10, 15, and 20 ethylene oxide units. We aimed to examine how these oleyl homologues interact with and subsequently impact the barrier properties of SC lipid bilayers at the molecular level. To our knowledge, this is the first molecular simulation study to examine the effects of both the methyl oleate and POE oleyl ethers on SC lipid bilayers. These findings provide important new insights into how common pharmaceutical and industrial coformulants influence the skin barrier.

## Methods

### Force Field Parameters

All simulations were performed using the GROMACS 2023.3 simulation package.^31–33^ The all-atom CHARMM36 force field^34^ was used due to its validated parameters for lipids, including ceramides.^35,36^ Water molecules were modeled using the TIP3P water model.^37^ Force field parameters for the oleyl homologues were generated using the automated CHARMM General Force Field (CGenFF) parameter assignment tool,^38,39^ as these molecules (with the exception of oleic acid) are not available in CHARMM36. All generated molecules had initial penalty scores of zero for both partial charges and bonded terms, and therefore warranted no further optimization. The generated GROMACS topology (.itp) files for oleic acid, methyl oleate and POE­(5, 10, 15 and 20) oleyl ethers are provided in the Supporting Information.

### Construction of Model SC Bilayers

Two approaches were used to construct the initial configurations of our systems: the CHARMM GUI multicomponent assembler and membrane builder,^40,41^ and PACKMOL.^42^ The CHARMM multicomponent assembler was used to generate bilayer systems with the oleyl homologues initially placed in the solvent layer, while PACKMOL was used to generate configurations in which the oleyl homologues were embedded within the bilayer.

### Bilayer Construction Using the CHARMM GUI

The CHARMM GUI multicomponent assembler was used to generate lipid bilayers consisting of an equimolar mixture of CER­[NP], C_24_ fatty acid and cholesterol, with 98 molecules of each lipid per leaflet, yielding a total of 588 lipids in a 10 × 10 nm bilayer patch. This system is hereafter referred to as the “control” and represents the lipid bilayer in a water environment. CER­[NP] was modeled with the most common saturated lipid tail lengths of 24 and 18 carbons. The CER­[NP] model from CHARMM36 features 2R,3S,4R stereochemistry on the phytosphingosine base (as shown in Figure 2). We note that this differs from the most abundant phytosphingosine stereochemistry (2S, 3S, 4R) found in nature, but we do not expect this to make a significant difference.^43^ This lipid composition was selected based on previous work showing that such mixtures reproduce the gel-phase dynamics and strong barrier functions characteristic of the SC lipids.^44^ Each model bilayer was solvated with a 5 nm water layer on either side, equating to ∼30,000 water molecules, and representing a fully hydrated SC lipid bilayer. Sixty molecules of a single oleyl homologue were randomly placed in the water layer. These systems were then simulated to assess whether the oleyl homologue molecules would spontaneously partition into the SC lipid bilayers.

### Bilayer Construction Using PACKMOL

Following the spontaneous insertion simulations, which confirmed that all modeled oleyl homologues could partition into the SC lipids, we generated a second set of systems in which these molecules were preinserted into the bilayer. This allowed us to study their effects without the transient local disruption caused by the insertion process. A 5 mol % concentration was chosen after the spontaneous insertion simulations showed that all oleyl homologues partition into the SC lipid bilayers up to a 5 mol % concentration. PACKMOL was used to construct these systems by adding 15 molecules of each oleyl homologue into each leaflet of the standard SC lipid bilayer. Position restraints for the headgroup and tail of each lipid species (CER­[NP], C_24_ fatty acid and cholesterol) were taken from the average atom positions of the lipid headgroups and terminal CH_3_ group for each lipid species, extracted from the spontaneous insertion simulations. For the oleyl homologues, the headgroup restraint was taken from the average position of the first headgroup oxygen and the tail restraint from the terminal CH_3_ group in the oleyl tail from successfully partitioned oleyl homologue molecules generated from the spontaneous insertion simulations. As with the CHARMM GUI bilayers, a 5 nm water layer was added to each side of the bilayer, equating to ∼30,000 water molecules per system. A representative SC lipid bilayer is shown in Figure S1.

### Simulation Protocol

In this study, we performed two sets of simulations, each repeated 3 times. In the first, each oleyl homologue was solvated in water in the presence of the SC lipid bilayer and simulated to determine whether it would spontaneously partition into the bilayer. In the second, the oleyl homologues were preinserted into the bilayer (15 molecules per leaflet, constituting a ∼5 mol % concentration with respect to the lipids) and simulated to determine their effects on bilayer structure and stability, without the transient disruption caused by partitioning. Repeats differed by starting configuration.

All simulations were performed at 303.15 K and 1 atm, using the v-rescale thermostat and c-rescale barostat, with coupling constants of 1 and 5 ps, respectively. Pressure was applied semi-isotropically along the *xy* and *z* axes. Solvent and bilayer atoms were coupled separately in the thermostat. In the spontaneous insertion simulations, oleyl homologues were assigned to the solvent thermostat, while in the preinserted systems, they were coupled with the bilayer. Nonbonded interactions were treated using a cutoff scheme for short-range interactions and particle-mesh Ewald (PME) for long-range electrostatics.^45^ A cutoff of 1.2 nm was applied to both van der Waals and short-range electrostatic interactions. For van der Waals interactions, a force-switching function was applied between 1.0 and 1.2 nm. Covalent bonds involving hydrogen atoms were constrained using the LINCS algorithm.^46^ Periodic boundary conditions were applied in all three spatial dimensions.

The same equilibration and production run protocols were used for both the CHARMM-GUI and PACKMOL-generated bilayers. Equilibration protocols were based on those provided by CHARMM-GUI. Systems were first energy-minimized using the steepest descent algorithm, followed by two NVT equilibration steps of 125 ps, each with a 1 fs time step, and with decreasing *xyz* position restraints from 1000 to 400 kJ mol^–1^ nm^–2^. These were followed by a series of four NPT equilibration steps of 250 ps each with a 2 fs time step, and with position restraints gradually reduced from 400 to 0 kJ mol^–1^ nm^–2^. Following this, production simulations were run for 500 ns using a 2 fs time step.

### Analysis

#### Lipid Bilayer Density

The lipid bilayer water density was calculated using the GROMACS tool gmx density. The density profiles are calculated using 200 slices along the bilayer normal. Average densities were calculated by taking the mean average across the three replicates. Error values were calculated as the standard error across the three replicates.

#### Area per Lipid

Area per lipid values were calculated using the Voronoi tessellation method as described by Lukat et al.^47^ A custom implementation of this algorithm was developed using the MDAnalysis Python package to extract and process the relevant lipid headgroup atom positions.^48^ The specific headgroup atoms used for the tessellation are given in Figure S2. Following the construction of the Voronoi cells, we identified those directly adjacent to each oleyl homologue molecule to examine how the packing of neighboring lipids was affected. This allowed us to characterize localized packing defects that may not be captured within the global average area per lipid. Welch’s *t*-test was used with the means from each of the three repeats to determine if changes in the area per lipid were statistically significant from the control.

#### Area Compressibility Modulus

The area compressibility modulus, *K*_A_, was calculated using eq 1, where *K*_A_ is the area compressibility modulus, *k*_B_ is the Boltzmann constant, *T* is temperature, *A*_L_ is the area per lipid, *n*_L_ is the number of lipids and *sA*_L_ is the standard deviation of the area per lipid.

1 $$K_{A}=\frac{2k_{B}T\left\langle A_{L}\right\rangle }{n_{L}{\left(sA_{L}\right)}^{2}}$$

The production simulations were divided into 10 50 ns segments and a *K*_A_ value calculated for each segment, following the approach described by Kapla.^49^ The values from each time segment and each simulation repeat were combined to calculate the mean and standard error. Welch’s *t*-test was used with the means from each of the three repeats to determine if changes in the area compressibility modulus were statistically significant from the control.

#### Solvent Accessible Surface Area

In order to calculate the solvent-accessible surface area (SASA), we used a rolling ball algorithm with a probe radius of 1.8 Å (approximating a water molecule) to generate a topological description of the bilayer surface. Lipid atoms from the upper leaflet were used to define the bilayer, and coordinates were sampled every 10 ps from the final 250 ns of the production simulations. The bilayer surface was discretized on a 2 Å × 2 Å grid in the *xy* plane. For each grid point, the highest *z*-coordinate lipid atom was selected, and a smoothed surface was generated using Gaussian smoothing and a point cloud constructed, representing the SASA.^50^ The Open3D python package^51^ was then used to perform a Poisson surface reconstruction on the point cloud to yield a watertight mesh describing the bilayer surface.^52^ We calculated the distance between each vertex of the mesh and the heavy atoms of the oleyl homologue headgroups. Mesh vertices located within 3.5 Å of any oleyl homologue atom were removed. The final surface area was then calculated using Open3D’s built-in method. Welch’s *t*-test was used with the means from each of the three repeats to determine if changes in the SASA were statistically significant from the control.

#### Water Permeability via Widom Particle Insertion

To assess the barrier properties of the SC lipid bilayers, we used the Widom particle insertion (WPI) method to calculate the excess chemical potential profile of water across the lipid bilayers as a function of the *z*-position of the water molecule relative to the bilayer normal.^53^ By evaluating changes in the chemical potential profile, we quantified the effect of oleyl homologue inclusion on the bilayer’s ability to act as a barrier to water permeation, which may correlate with transepidermal water loss. The excess chemical potential was calculated using eq 2, in 2 Å-thick slices along the *z*-axis, where μ_ex_ is the excess chemical potential, *k*_B_ is the Boltzmann constant, *T* is the temperature and ψ_*i*_ is the interaction energy of an inserted water molecule with the rest of the system.

2 $$\mu_{ex}=-k_{B}T\ln\left(\langle \exp\left(-\frac{\psi_{i}}{k_{B}T}\right)\right)\rangle )$$

The resulting position-dependent free energy profile for water insertion is analogous to a potential of mean force obtained via umbrella sampling, yet was computed at a fraction of the computational cost.

We used the GROMACS “tpi” module to perform the test particle insertions. We introduced a patch to the “tpi.cpp” source code to enable sampling within a slab of specified thickness along the *z*-axis. To avoid artifacts near the box edges, we introduced an additional condition to exclude trial coordinates within 1.2 nm of the box edge, matching the short-range Coulomb cutoff distance. The patched tpi.cpp code is available via the Supporting Information. We performed a series of tests to validate our implementation of WPI, which are described in Section 1.2 of the Supporting Information.

WPI is computationally inexpensive but suffers from poor convergence in dense systems due to the low probability of successful insertions.^54^ While more advanced alternatives exist, such as the cavity insertion method,^55^ we addressed this challenge by increasing our sampling density and simulation length. Convergence was verified by monitoring the plateau of chemical potential values over sampling time within each slice. Presented profiles were taken as the mean from three simulation replicates. Error in the profiles was calculated as the standard error of the final free energy profile for each of the three replicates. Final presented profiles are symmetrized by averaging around zero and have a weak sliding window smoothing applied, where each point becomes the mean of itself, and the previous and next free energy value.

Test particle insertions were performed on the final 250 ns of the production simulations, totalling 25,000 frames. Within each frame, 50,000 random insertions were attempted inside the sampled 75 Å × 75 Å × 2 Å slab (∼5 insertions per Å ^3^). Each insertion was repeated 500 times, where the water molecule was randomly rotated and displaced (no more than 0.25 Å) about its initial test position. Sampling was performed on slabs 1 Å apart, with a slab thickness of 2 Å, from 1.5 to 13.5 nm along the *z*-axis, resulting in 120 test particle insertion calculations along the *z*-axis. Interaction energies were calculated using a van der Waals cutoff of 1.2 nm, with a force-switch applied between 1.0 and 1.2 nm. Electrostatic interactions were treated using the PME method with a real-space cutoff of 1.2 nm. We found that PME was essential for obtaining physically meaningful chemical potential profiles (as discussed in Section 1.2 of the Supporting Information).

#### Hydrogen Bonding Networks

It is well established that intermolecular hydrogen bonding plays a crucial role in maintaining lipid bilayer structural stability.^56,57^ In addition to direct lipid–lipid hydrogen bonds, lipid molecules can also engage in water-mediated interactions that form extended interfacial hydrogen bond networks.^58^ These networks span the bilayer plane to connect lipids laterally and can also span across stacked bilayers, contributing to membrane cohesion in both single and multilamellar systems.^59,60^ The addition of exogenous compounds, such as the oleyl homologues, has the potential to alter both direct and water-mediated hydrogen bonding, thereby impacting bilayer structure and stability.

Average numbers of direct lipid–lipid and lipid–water hydrogen bonds were calculated using the GROMACS “hbond” module. To analyze network connectivity, hydrogen bond data were extracted every 1 ns from the final 250 ns of each production simulation. For each frame, hydrogen bond donors and acceptors were identified using MDAnalysis, and water molecules located more than 1.0 nm from the bilayer surface were excluded.

Hydrogen bond pairs were stored as an undirected graph, with nodes representing unique lipid or water molecules and edges corresponding to hydrogen bond connections. A series of depth-first searches were performed on this graph, with increasing maximum path lengths, to calculate the number of lipid–lipid connections mediated by the interfacial hydrogen bonding network.

Additionally, we used the python package igraph^61^ to calculate network properties including the average transitivity and eigenvector centrality. The transitivity is a measure of the network’s tendency to form closed triplets (triangles), or, in our case, three molecules hydrogen bonded to each other.^62^ An example of a closed triplet hydrogen bond cluster can be seen in Figure S4. The eigenvector centrality is a measure of the average influence of the network, or alternatively, how well connected the nodes are based on the connectivity of their neighbors. Decreases in the centrality imply a loss of cross-linking or fragmentation of the network into smaller, disconnected subnetworks.^63^

Welch’s *t*-test was used with the means of the hydrogen bond network connections and network properties from each of the three repeats to determine if changes in the hydrogen bond network were statistically significant from the control.

#### Lipid Order Parameters

Lipid deuterium order parameters provide a measure of how well the lipid tails are aligned with the bilayer normal (*z*-axis). Perfectly extended lipid tails aligned along the bilayer normal yield an order parameter, *S*_CD_, of 1.0, while isotropically arranged (i.e., disordered) lipid tails yield order parameters near 0.0, and lipids aligned perpendicular to the *z*-axis produce negative values, approaching a minimum of −0.5. The order parameter was calculated using eq 3, where θ is taken as the angle between the *z*-axis and the bond vector connecting carbon atoms C_*n*–1_ and C_*n*+1_ and *S*_CD_ is the C_*n*_ deuterium order parameter.

3 $$S_{CD}=\frac{1}{2}\left(3\cos^{2}\theta -1\right)$$

To account for localized effects of oleyl homologue inclusion, we calculated the order parameter of each CER­[NP] and C_24_ fatty acid within our SC lipid bilayers and, using the Voronoi tessellation map, identified the CER­[NP] and C_24_ fatty acid molecules immediately adjacent to the oleyl homologues and used these to calculate average localized order parameters. Welch’s *t*-test was used with the means from each of the three repeats to determine if changes in the lipid order parameters were statistically significant from the control.

#### Cholesterol Tilt

Traditional order parameters cannot be calculated for cholesterol due to its rigid ring structure. Instead, we defined a vector spanning the rigid cholesterol ring system and calculated the angle between this vector and the bilayer *z*-axis. To evaluate whether oleyl homologues influenced cholesterol orientation, we calculated both global and localized tilt values. Localized values were obtained by identifying cholesterol molecules directly adjacent to the oleyl homologues using the Voronoi tessellation maps. We then calculated the average cholesterol tilt angle and variance for these adjacent cholesterol molecules. Welch’s *t*-test was used with the means from each of the three repeats to determine if changes in the cholesterol tilt were statistically significant from the control.

## Results

### Spontaneous Insertion of Oleyl Homologues

Initial simulations were performed to test the ability of oleyl homologues to spontaneously partition into the SC lipid bilayers. Figure 4 shows representative results for oleic acid and POE(15) over a 500 ns simulation. In both systems, the molecules initially cluster on the bilayer surface, followed by gradual insertion into the bilayer. The insertion process involved association of the oleyl homologue headgroups with the lipid headgroups, followed by rotation and partitioning of the hydrophobic tails into the bilayer interior. In the case of oleic acid, all 60 (30 per leaflet) molecules were fully incorporated within 250 ns. In contrast, insertion of POE(15) occurred more slowly, with the formation of larger clusters on the surface of the bilayer appearing to hinder the partitioning process. After 500 ns, POE(15) and (20) were still partitioning, however the rate at which this occurred became slow across our simulation time scale. Simulation snapshots from each oleyl homologue spontaneous insertion system are available in Figure S5.

**Figure 4:**
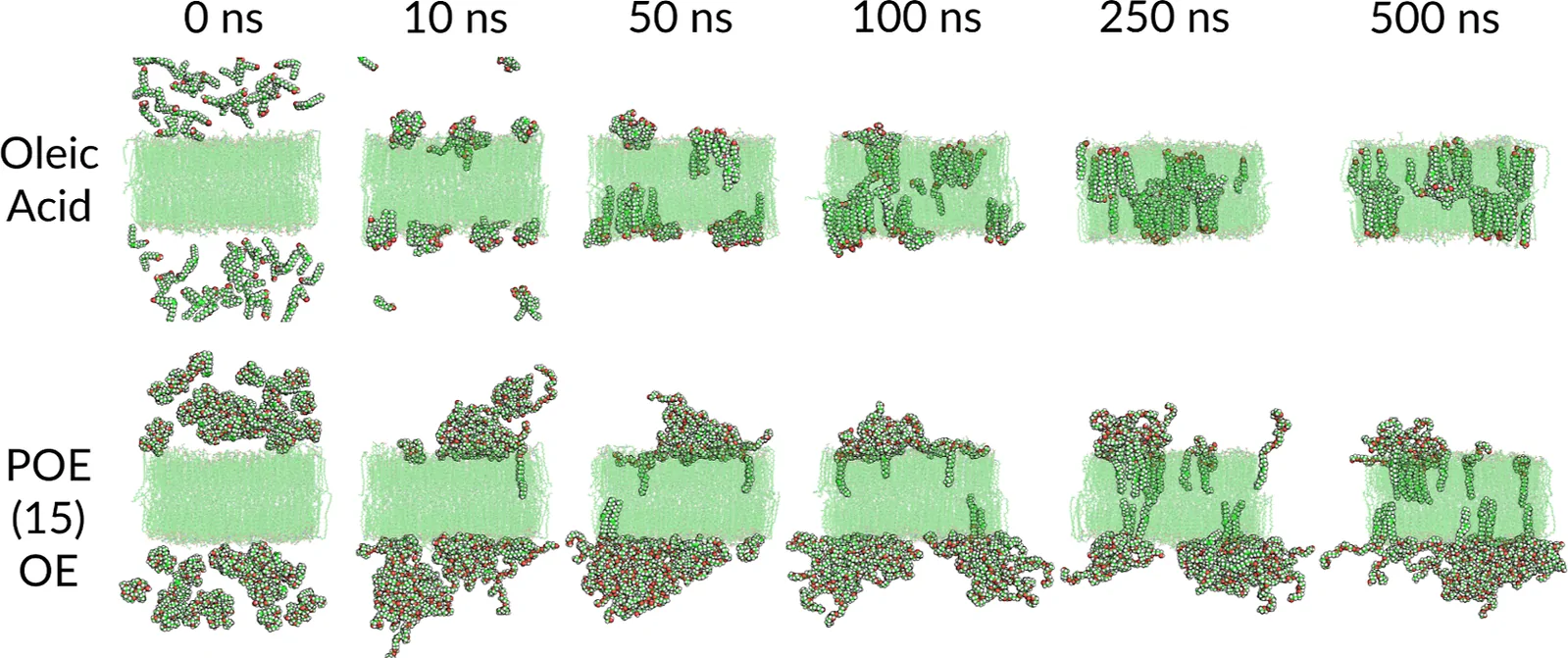
Representative simulation snapshots showing the spontaneous insertion of oleic acid and POE(15) oleyl ether into SC lipid bilayers over 500 ns. Snapshots were generated using PyMOL.^66^ Bilayer lipid molecules are shown as green lines, oleyl homologues are shown in space-filling representation and water molecules are omitted for clarity.

We observe two distinct partitioning behaviors. Incorporation occurred both via aggregate formation followed by partitioning, and via direct partitioning of individual molecules. For oleic acid and methyl oleate, incorporation was rapid and occurred via both aggregate-mediated and monomeric routes. As the POE tail was increased in length, monomeric insertion occurred less frequently, as the formation of aggregates and partitioning from these aggregates into the membrane dominated. The modeled concentration of each oleyl homologue in solution was 0.1 mol. This is substantially higher than the reported critical micelle concentrations for this homologous series (6 μmol for oleic acid, 0.94 mmol for POE(10), and 0.265 mmol for POE(20)).^64,65^ The results demonstrate that partitioning occurs spontaneously above the CMC. Our observations of the molecules inserting as monomers suggest that partitioning is also likely to persist at concentrations below the CMC of the oleyl homologues.

The partitioning of the oleyl homologues from solution into the membrane caused some temporary disruption to the membrane lipids, which allowed some water molecules to enter the membrane alongside the inserted oleyl homologues. As we did not observe water molecules inside the membranes with the oleyl homologues preinserted (results discussed below), we assume that this disruption is short-lived and does not contribute to the mechanism of permeability enhancement on realistic time scales.

### Effects of Oleyl Homologue Inclusion on the Structural and Mechanical Properties of the Bilayers

Having seen that all modeled oleyl homologues were able to partition spontaneously into the SC lipid bilayers on relatively short time scales (<500 ns), we generated a new set of systems with 5 mol % of each homologue preinserted into the bilayer (15 molecules per leaflet). This approach ensured a uniform concentration across the systems and eliminated transient artifacts caused by spontaneous insertion, which may have impacted our results.

First, we examined the area compressibility modulus, *K*_A_, as a quantitative indicator of bilayer deformability (Figure 5). In the control system without any oleyl homologues, we observed a high *K*_A_ value of 5000 mN m^–1^, consistent with previous simulations of model SC lipid mixtures, which report values of ∼4000 mN m^–1^ at 360 K, with the difference assumed to be due to our lower simulation temperature of 303.15 K.^67,68^ This high area compressibility modulus has been attributed to the strong lateral hydrogen bonding network present in the SC lipid bilayers. Following the addition of oleic acid and methyl oleate, *K*_A_ decreased to 4300 mN m^–1^. Interestingly, the inclusion of POE oleyl ethers up to 15 ethylene oxide units produced a modest recovery in *K*_A_, before a further decrease was observed for the POE(20) system. Despite this recovery, all systems containing oleyl homologues exhibited lower *K*_A_ than the control, suggesting all of the oleyl homologues disrupt the lipid–lipid interactions in the bilayer to some extent. The changes in area compressibility modulus for oleic acid, methyl oleate and POE(20) were statistically significant (*p* < 0.05), and the changes for POE(5), POE(10) and POE(15) were weakly significant (*p* < 0.1).

**Figure 5:**
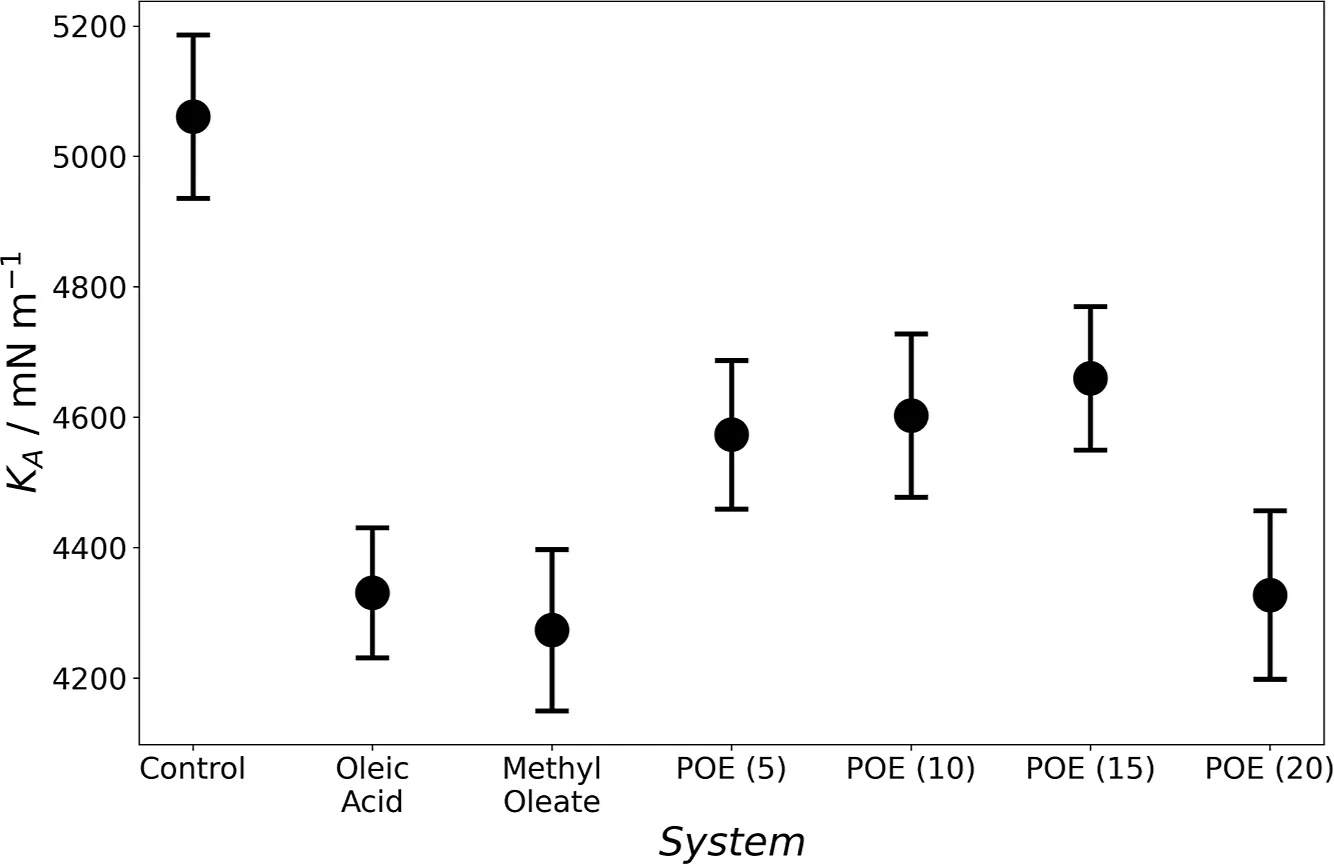
Area compressibility modulus of SC lipid bilayers with 5 mol % oleyl homologue inclusion.

In addition to the area compressibility modulus, we analyzed the area per lipid to examine how oleyl homologues impact lipid lateral packing. Figure 6 shows the average area per lipid of each individual bilayer component from each modeled system. Addition of oleyl homologues had little effect on the bulk average area per lipid values, with minor changes observed for the CER­[NP], C_24_ fatty acid and cholesterol areas compared to the control system. However, a notable increase in the standard error of the mean was observed in systems containing methyl oleate and POE oleyl ethers. This increase coincided with an increase in the average area per lipid of the oleyl homologues themselves from 26 Å ^2^ in the oleic acid system to ∼30 Å ^2^ for methyl oleate and the POE oleyl ethers. The oleyl homologues were found to have no statistically significant effect on the bulk average area per lipid values (*p* > 0.05) for all systems.

**Figure 6:**
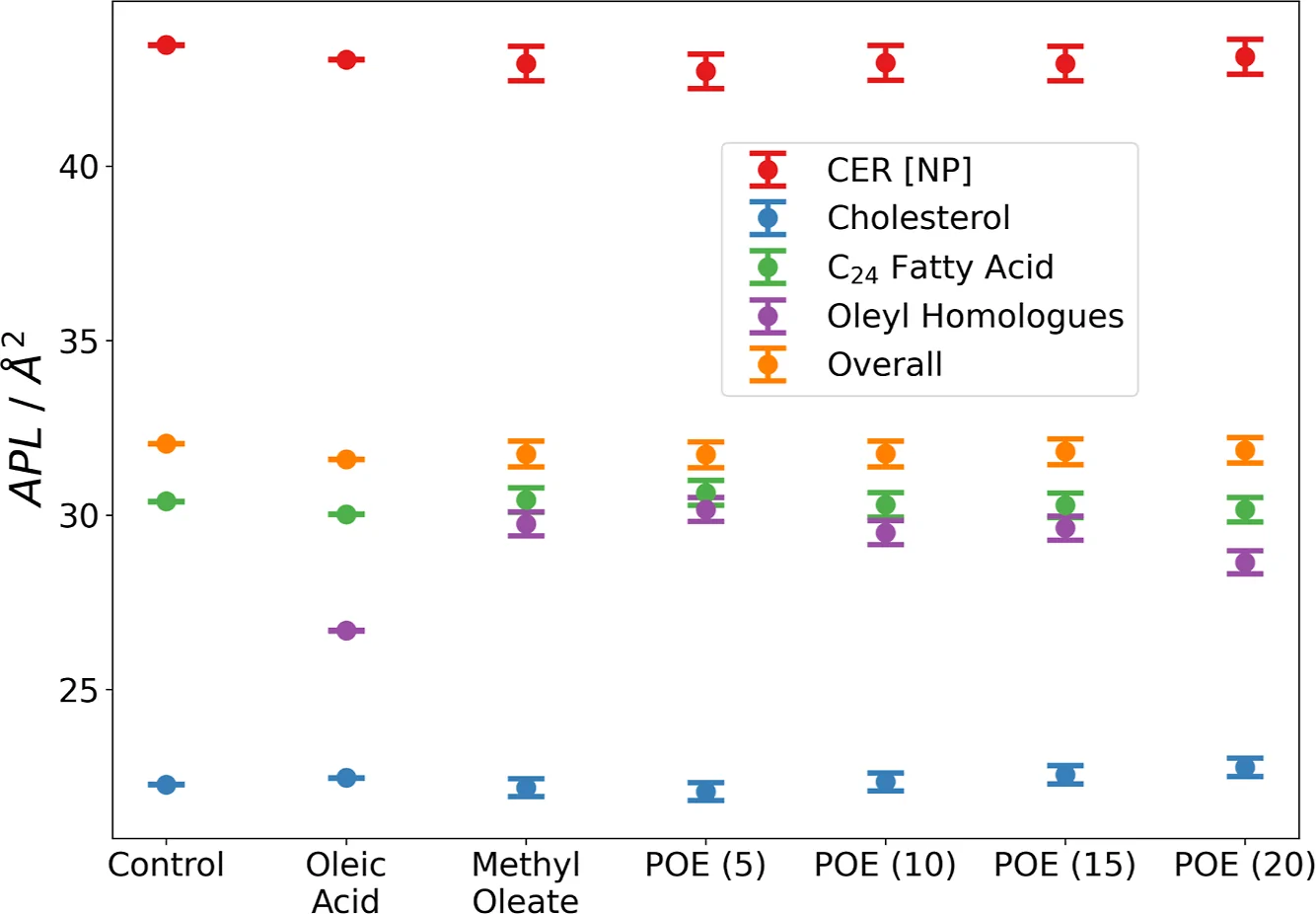
Area per lipid values for each bilayer component in the modeled systems. Error bars represent the standard error of the mean across repeat simulations.

In addition to the bulk average area per lipid, the area of lipids directly adjacent to oleyl homologues was computed in order to ascertain whether these molecules induce localized packing defects. Figure 7 shows the average percentage increase in the cholesterol area per lipid when directly adjacent to an oleyl homologue, compared to the bulk average cholesterol area per lipid. On average, adjacent cholesterol molecules exhibited a ∼10% increase in the area per lipid. This effect was consistent across all the oleyl homologues, with variation remaining within the calculated error. Overall, these findings suggest that oleyl homologues disrupt the lateral packing of neighboring cholesterol molecules. The consistent ∼10% increase across oleyl homologues, despite differences in headgroup chemistry, implies that the oleyl tail is the governing factor driving local cholesterol disordering. Local changes in the cholesterol area per lipid were found to be statistically significant (*p* < 0.05) for the oleyl ethers, while the changes in the oleic acid and methyl oleate system were found to be statistically insignificant (*p* > 0.05).

**Figure 7:**
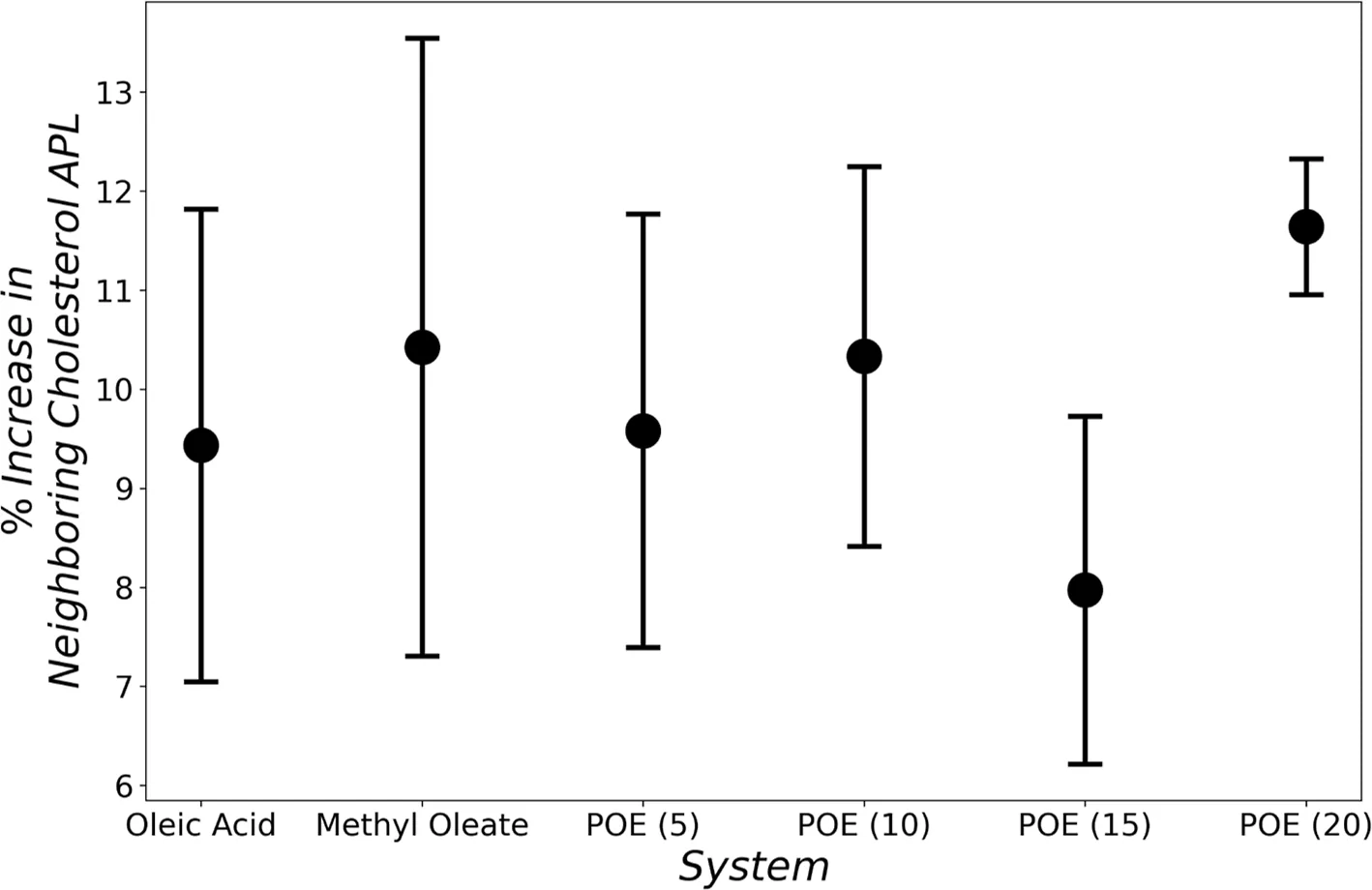
Average increase in area per lipid of cholesterol molecules directly adjacent to oleyl homologues compared to the bulk average cholesterol area per lipid.

In addition to cholesterol, we examined the local area per lipid values for the CER­[NP] and the C_24_ fatty acid. As shown in Figure S6, local CER­[NP] area per lipid values exhibited minimal changes (<3%) across all systems. In contrast, the C_24_ fatty acid local area per lipid showed a more pronounced response. As shown in Figure 8, local area per lipid remained largely unchanged in the presence of oleic acid and methyl oleate; however, addition of the POE oleyl ethers led to a 5% increase in the local area per lipid, increasing gradually with lengthening POE headgroup. This trend demonstrates an increasing degree of packing disruption with increasing POE headgroup length. Changes in the localized ceramide area per lipid were found to be significant (*p* < 0.05) for the oleyl ethers, changes in the fatty acid area per lipid were found to be statistically insignificant (*p* > 0.05) for all modeled systems.

**Figure 8:**
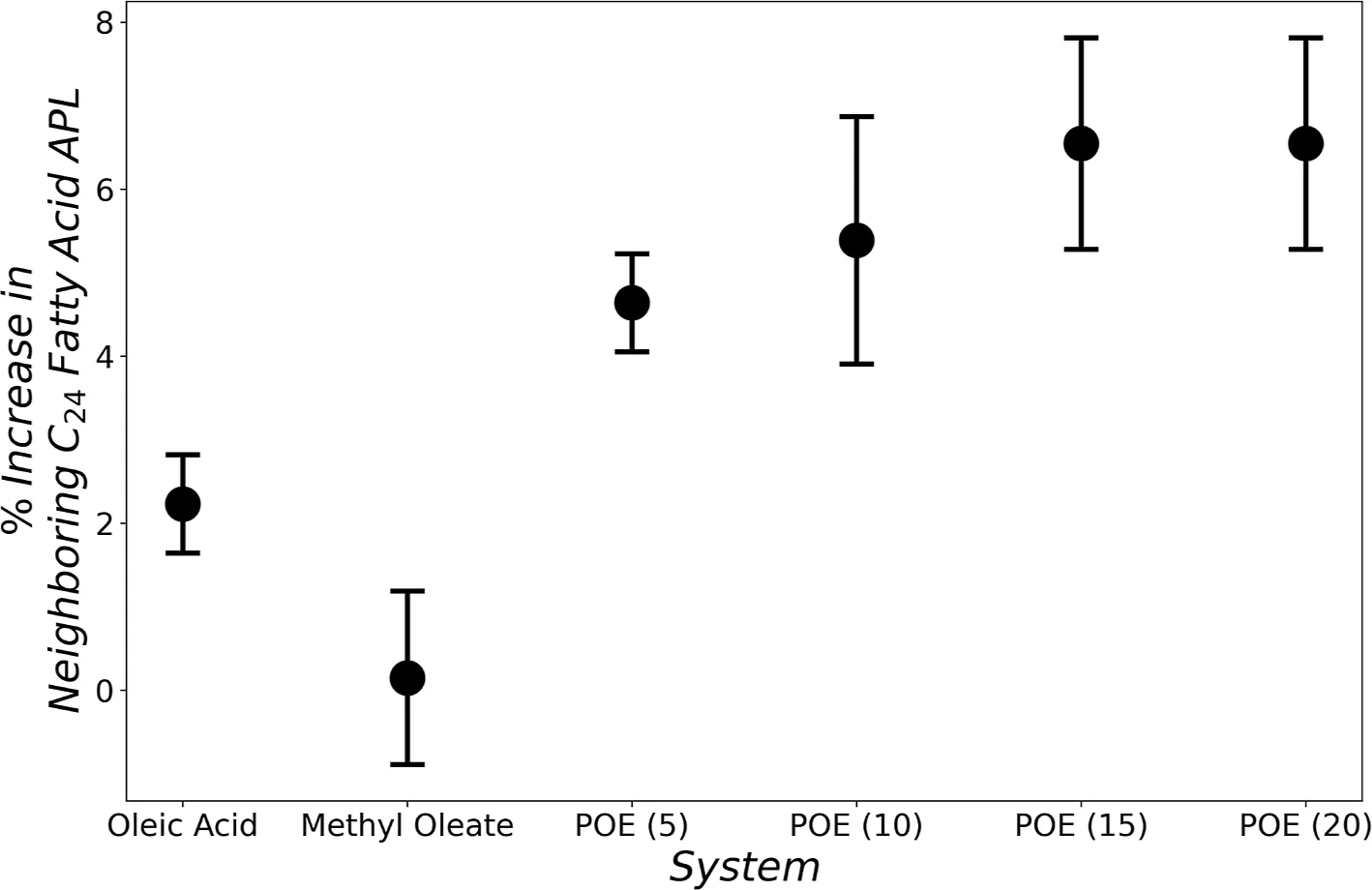
Average increase in area per lipid of C_24_ fatty acid molecules directly adjacent to oleyl homologues compared to the bulk average C_24_ fatty acid area per lipid.

The structural organization and packing efficiency of the bilayer lipids were further assessed by calculating deuterium order parameters. Figure 9 shows the bulk average order parameters for both the CER­[NP] tails and the C_24_ fatty acid, as well as the order parameters of those lipids located adjacent to an oleyl homologue. Bulk average values from our control system containing no oleyl homologues are also shown for comparison. For the CER­[NP] long tail, order parameters remained largely unchanged, indicating that this chain retains its ordered structure across all systems. In contrast, the short tail of CER­[NP] exhibited a pronounced decrease in order at carbons 2 and 3 upon addition of any oleyl homologue. This effect was most significant in the presence of oleic acid. Notably, carbons 2 and 3 correspond to the hydroxyl-bearing carbons in the CER­[NP] headgroup, suggesting that interaction of oleyl homologue headgroups with these hydroxyl groups may mediate local disorder. Figure S7 in the Supporting Information highlights carbon 2 and 3 of the CER­[NP] short tail. Finally, for the C_24_ fatty acid, reduced order was observed from carbon 9 onward. This disruption became more pronounced with increasing POE headgroup length, with the greatest effect observed for the POE(20) oleyl ether. In summary, the addition of oleyl homologues induces disorder in distinct regions of the lipid tails. In CER­[NP], the effect is strongest near the headgroup and most pronounced with oleic acid. In contrast, the C_24_ fatty acid becomes increasingly disordered further along the hydrocarbon chain, particularly with larger POE headgroups.

**Figure 9:**
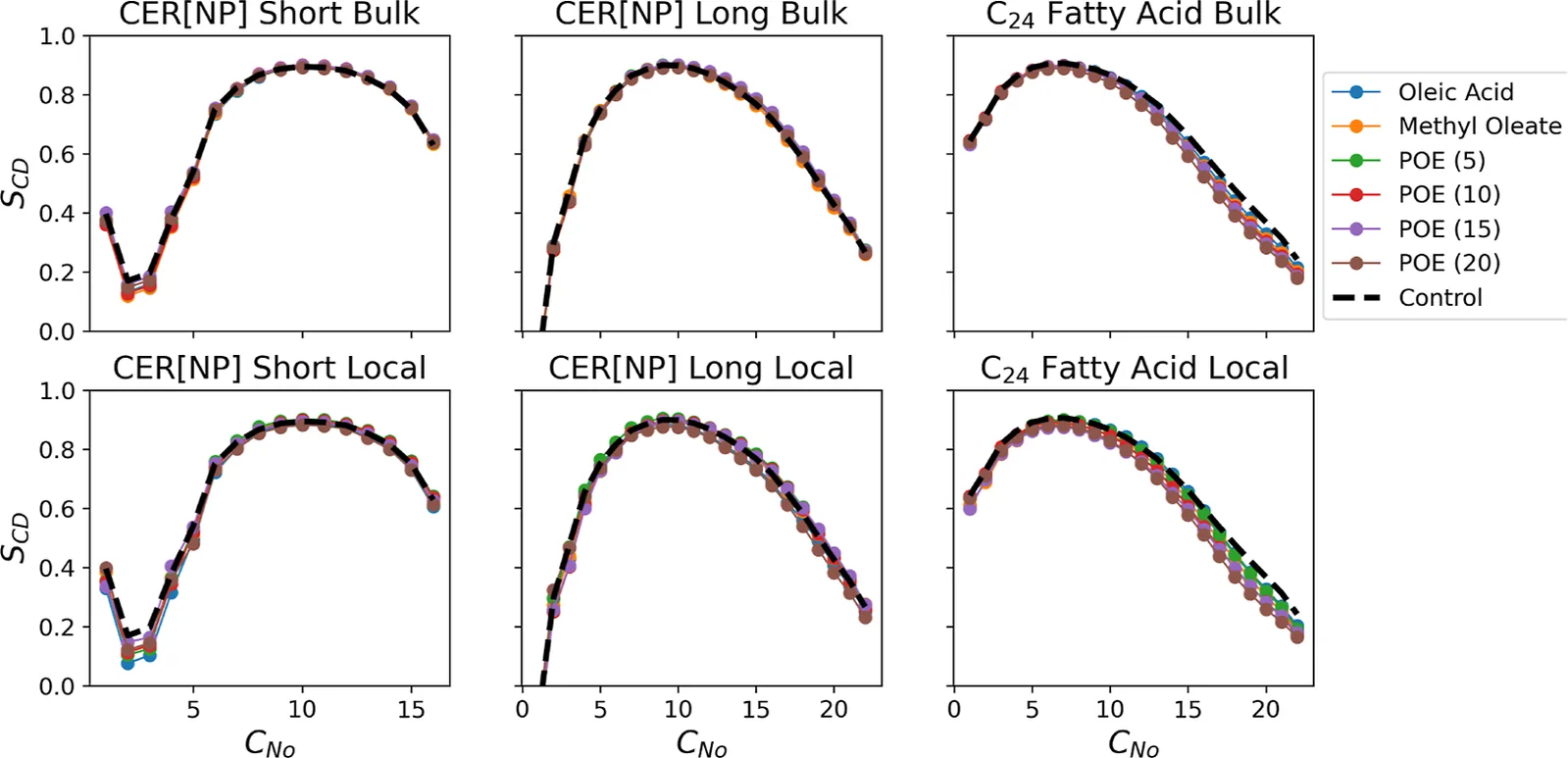
Deuterium order parameters for the short and long tails of CER­[NP] and the C_24_ fatty acid, shown for bulk ensembles and lipids adjacent to oleyl homologues.

Following statistical analysis, the observed moderate decrease in the ceramide short chain headgroup order from carbons 1 to 4 induced by oleic acid, methyl oleate and POE(5) oleyl ether was found to be statistically significant (*p* < 0.05) in both the bulk and local order parameters. In the long ceramide tail, there were no statistically significant differences between the control and oleyl homologue systems in the bulk profiles, but in the local profiles, the moderate decrease in lipid order induced by oleic acid and POE(20) oleyl ether from carbon 9 onward was found to be statistically significant (*p* < 0.05). Finally, for the fatty acid chains, the moderate decrease in order observed in both the bulk and local order profiles was found to be statistically significant (*p* < 0.05) in all but the oleic acid system in the bulk profiles, with little statistical significance from the local profiles.

Although we cannot calculate order parameters for cholesterol, the tilt of cholesterol with respect to the bilayer normal (*z* axis) provides a complementary measure of structural organization and packing. In Figure 10, we compare the average tilt of cholesterol molecules adjacent to oleyl homologues with the control systems. The average cholesterol tilt remained consistent across all systems, ranging between 10 and 12 degrees. However, the addition of the oleyl homologues leads to a marked increase in tilt angle variance from 1 to ∼7 degrees. This suggests that while the average orientation is unchanged, the conformational freedom of cholesterol increases in the presence of oleyl homologues, indicating local packing disruption. Statistical analysis shows that changes in the average cholesterol tilt are not significant as expected, but the change in cholesterol tilt variance is significant for all oleyl homologue systems (*p* < 0.05).

**Figure 10:**
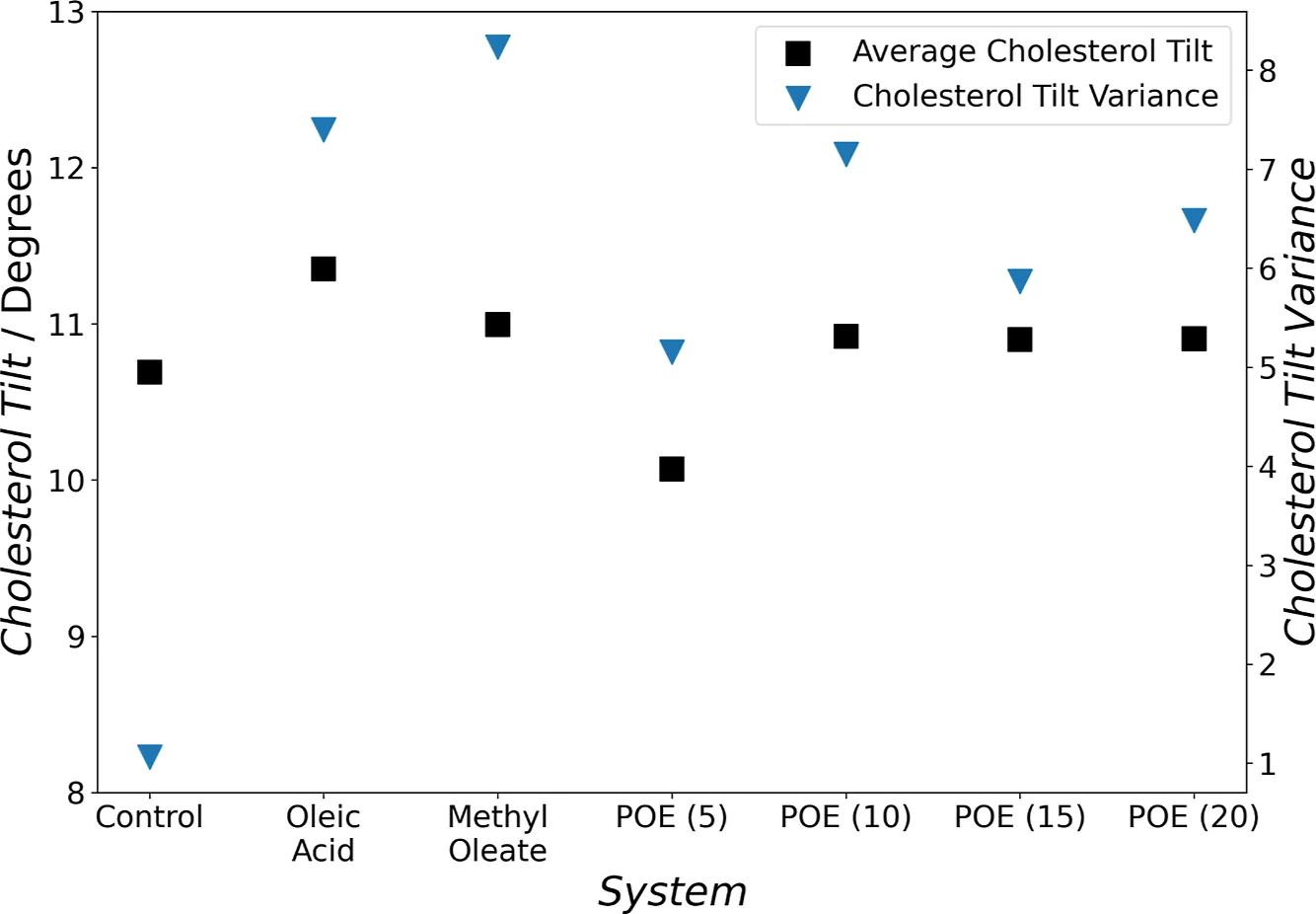
Average tilt angle and variance of cholesterol molecules adjacent to oleyl homologues, compared with the bulk average cholesterol tilt in the control system.

### Effects of Oleyl Homologue Inclusion on the Bilayer Interface

Interatomic interactions between lipid headgroups, particularly hydrogen bonding, are integral to maintaining bilayer structural stability. Extended hydrogen bonding networks, propagated through interfacial water, are important in supporting the barrier properties of the SC. Figure 11 shows how the interfacial hydrogen bonding network changes in the presence of the different oleyl homologues. We track the number of lipid–lipid connections mediated by hydrogen bond bridges of increasing length. Here, the bridge length denotes the number of water-mediated hydrogen bonds (with 0 being a direct lipid–lipid hydrogen bond). The *x*-axis indicates bridge length and the *y*-axis shows the average number of such connections per leaflet. The addition of oleic acid had a minimal effect on the hydrogen bond network compared to the control SC lipid bilayer. In contrast, the addition of methyl oleate and POE oleyl ethers resulted in a pronounced decrease in lipid–lipid connectivity. Specifically, the number of bridges of length 2 decreased from 400 to 370, length 3 from 590 to 530 and length 4 from 900 to 800. Among the homologues tested, POE oleyl ethers had the greatest disrupting effect, with longer POE chains associated with greater disruption to the hydrogen bond network. Following statistical analysis, we found that oleic acid had no statistically significant effect on the hydrogen bond network (*p* > 0.05). We determined that the decrease in hydrogen bond network strength at bridge lengths 3 and 4 in all but the oleic acid system was statistically significant (*p* < 0.05).

**Figure 11:**
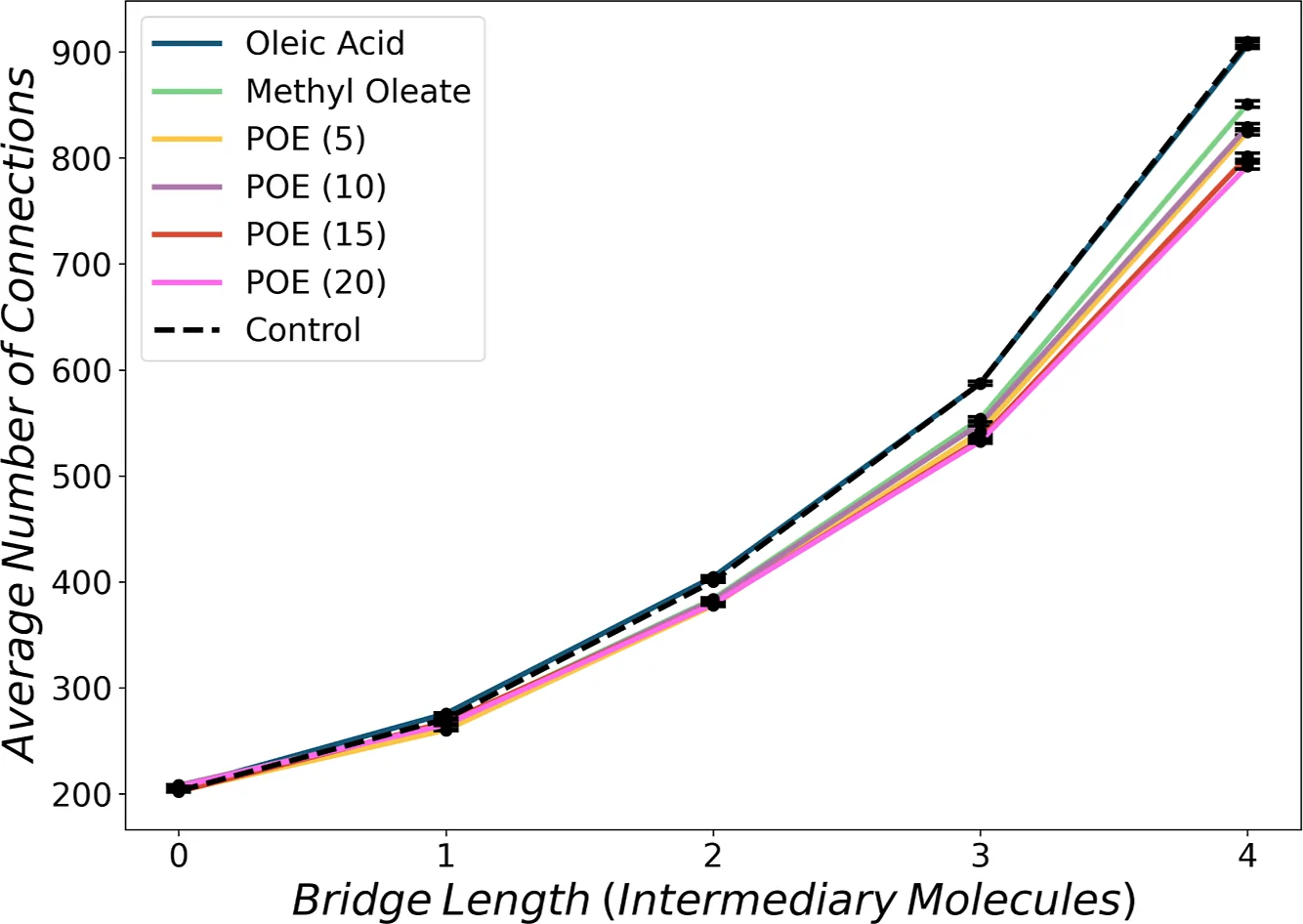
Number of water-mediated hydrogen bond bridges connecting lipid molecules across the SC lipid bilayer interfaces.

Figure 12 shows the effects of oleyl homologues on the hydrogen bond network transitivity and eigenvector centrality. The addition of oleic acid and methyl oleate had little effect on the network properties. In contrast, insertion of POE oleyl ethers with progressively longer POE chains led to a marked decrease in network connectivity. Specifically, the addition of POE(20) led to a 30% decrease in both network transitivity and eigenvector centrality. These reductions indicate that the interfacial hydrogen bond network becomes progressively less interconnected in the presence of longer POE headgroups, reflecting increased disruption at the bilayer surface. Following statistical analysis, the decreases in eigenvector centrality and transitivity in the POE oleyl ether systems were found to be statistically significant (*p* < 0.05).

**Figure 12:**
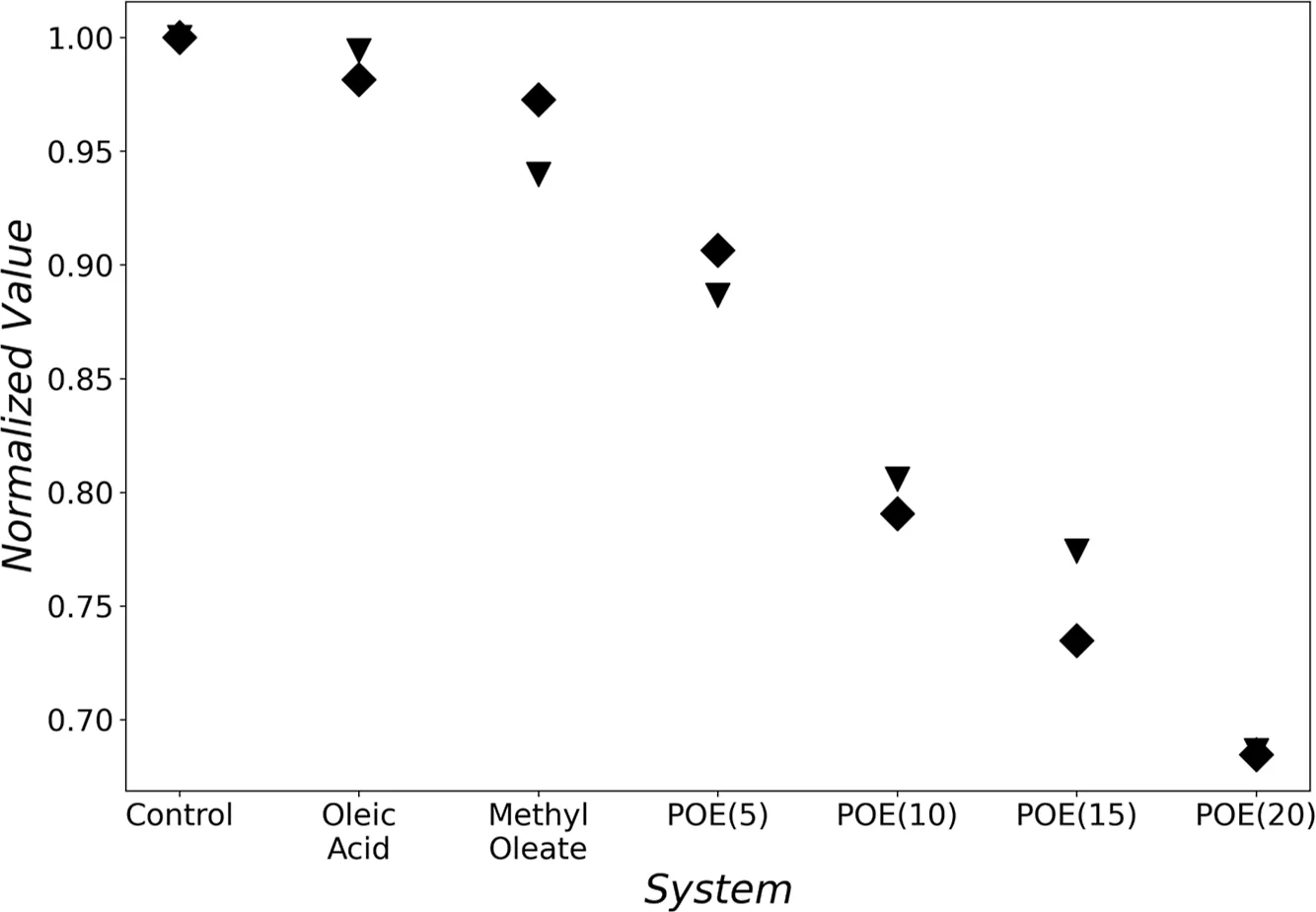
Transitivity (triangles) and eigenvector centrality (diamonds) of the interfacial hydrogen bond network, comparing control and oleyl homologue-containing systems.

In addition to the extended hydrogen bond network, we examined the number of direct hydrogen bonds formed between bilayer lipids and water molecules (Figure 13). Numbers of Cholesterol–Cholesterol and C_24_ Fatty Acid - C_24_ Fatty Acid hydrogen bonds are negligible and have been omitted from the figure for clarity. The complete hydrogen bond numbers for each system are available in the Supporting Information (Table ST1). The addition of oleic acid had no significant effect on lipid–lipid or lipid–water hydrogen bonds. However, as we move along the series of oleyl homologues from methyl oleate to POE oleyl ethers with increasing headgroup length, the number of hydrogen bonds formed between bilayer lipids and interfacial water molecules progressively decreased. Overall, inclusion of oleyl homologues, with the exception of oleic acid, disrupted lipid–water hydrogen bonding at the bilayer interface. This effect was most pronounced for the POE homologues with longer headgroups, suggesting that bulkier headgroups, such as the POE(20) headgroup, increasingly hinder the ability of interfacial water to interact with lipid headgroups.

**Figure 13:**
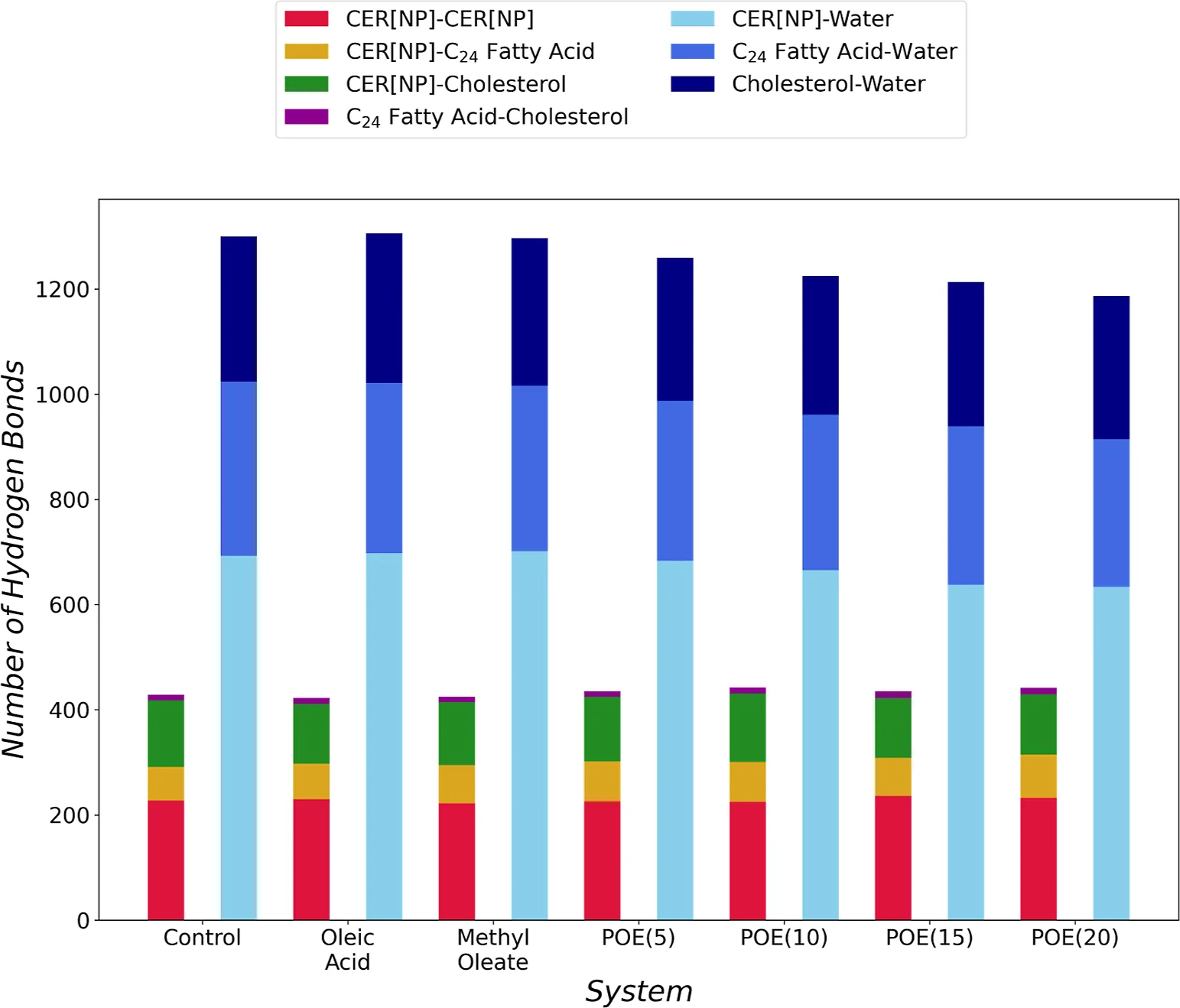
Number of lipid–lipid and lipid-solvent hydrogen bonds in the control and oleyl homologue systems.

The weakening of the hydrogen bond network implies some degree of bilayer surface disruption mediated by methyl oleate and POE oleyl ethers. To investigate this further, we calculated the SASA of a single bilayer leaflet (Figure 14). The addition of oleic acid or methyl oleate had a minimal effect on the SASA. However, the addition of POE oleyl ethers led to a significant and progressive reduction in the SASA. As the POE chain length increased, the average SASA decreased while its variance increased. This effect culminated in a 30% decrease in the SASA upon inclusion of the POE(20) oleyl ether. Figure 15 shows the smoothed mesh representation of the SASA for the final frame of a POE(20) simulation. Regions of the surface that are not accessible to water appear as gaps in the mesh. When the positions of the POE(20) molecules are overlaid, these gaps are seen to correspond with the location of the POE chains. The POE chains lie broadly flat on the bilayer surface, blocking access to the underlying lipid headgroups and thereby preventing interfacial solvent molecules from approaching, which explains the observed reduction in SASA. All observed changes in the solvent accessible surface area were found to be statistically significant (*p* < 0.05).

**Figure 14:**
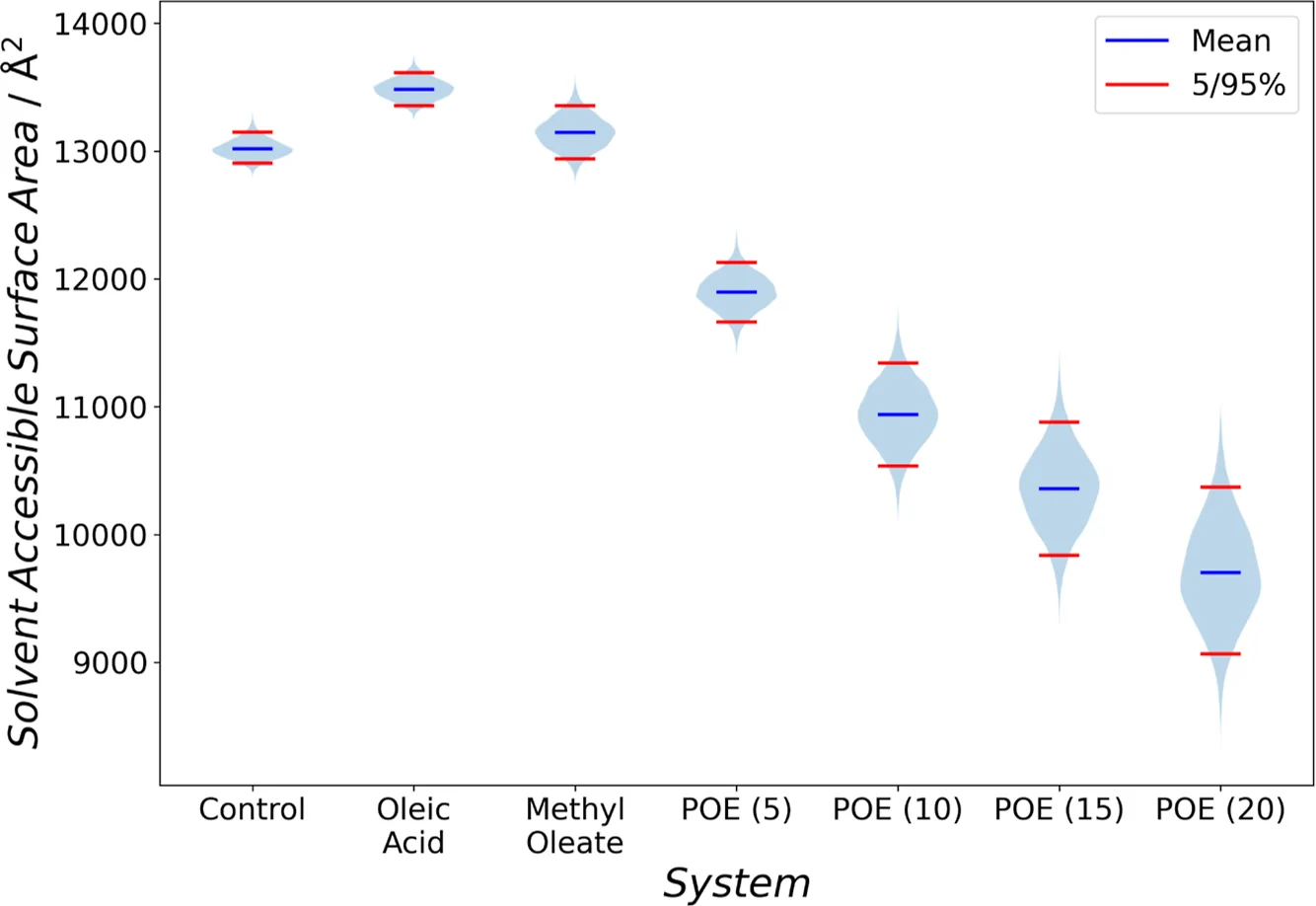
SASA in the control and oleyl homologue systems. The 95% confidence interval is marked in red, and the average values are shown in blue.

**Figure 15:**
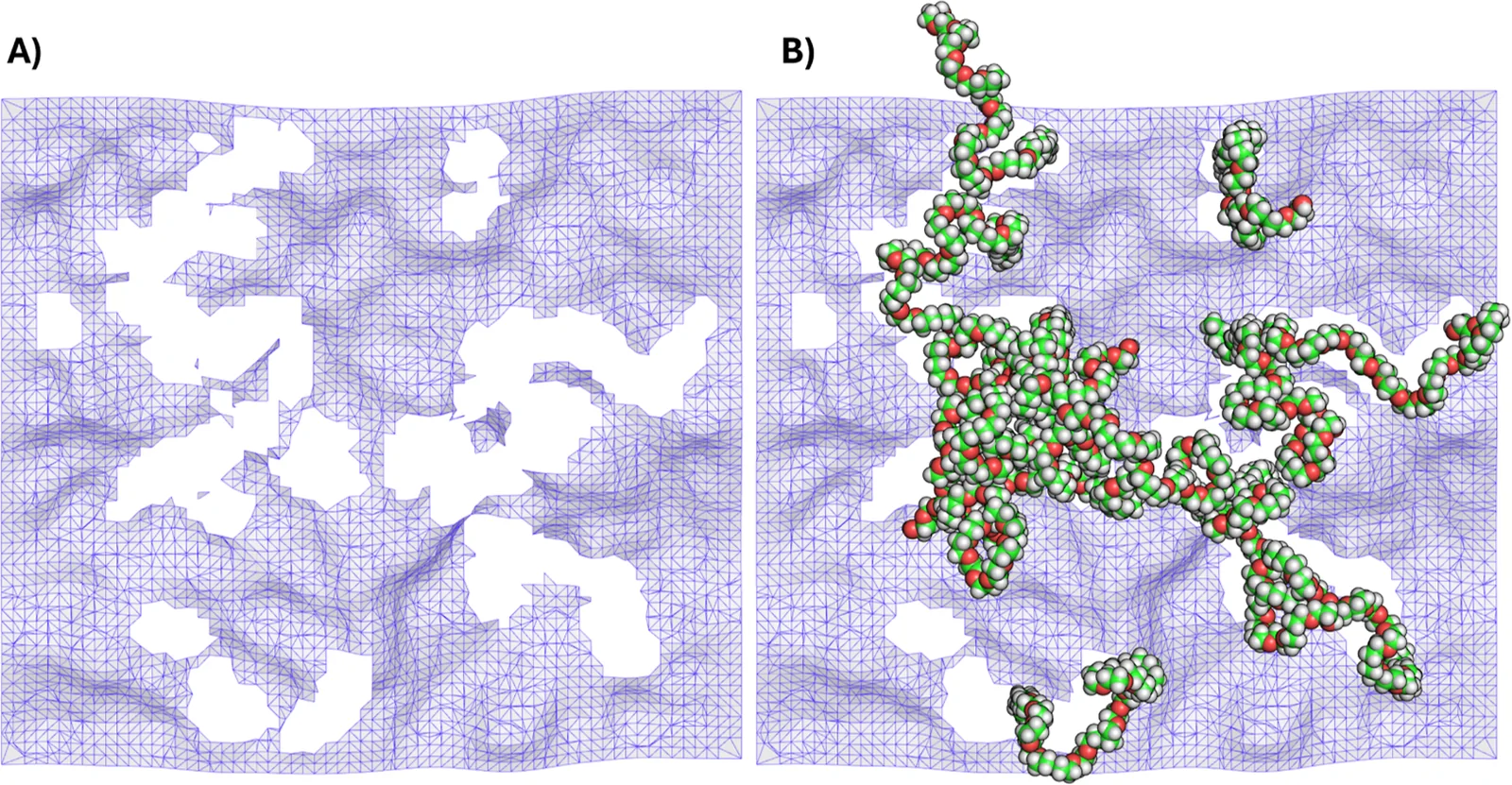
(A) SASA mesh of SC -lipid bilayer in the POE(20) oleyl ether system; (B) locations of POE(20) oleyl ethers relative to the mesh.

Additionally, we also calculated the water density profiles centered on the center of mass of the SC lipid membrane. Figure 16 shows the calculated water density profiles across the SC membrane for each modeled system. Oleic acid and methyl oleate show little deviation from the control system. As the POE oleyl ether headgroup increases in length, the water density on the membrane surface is decreased. No additional water permeation over the control is observed in any of the simulated systems within the simulated time scales.

**Figure 16:**
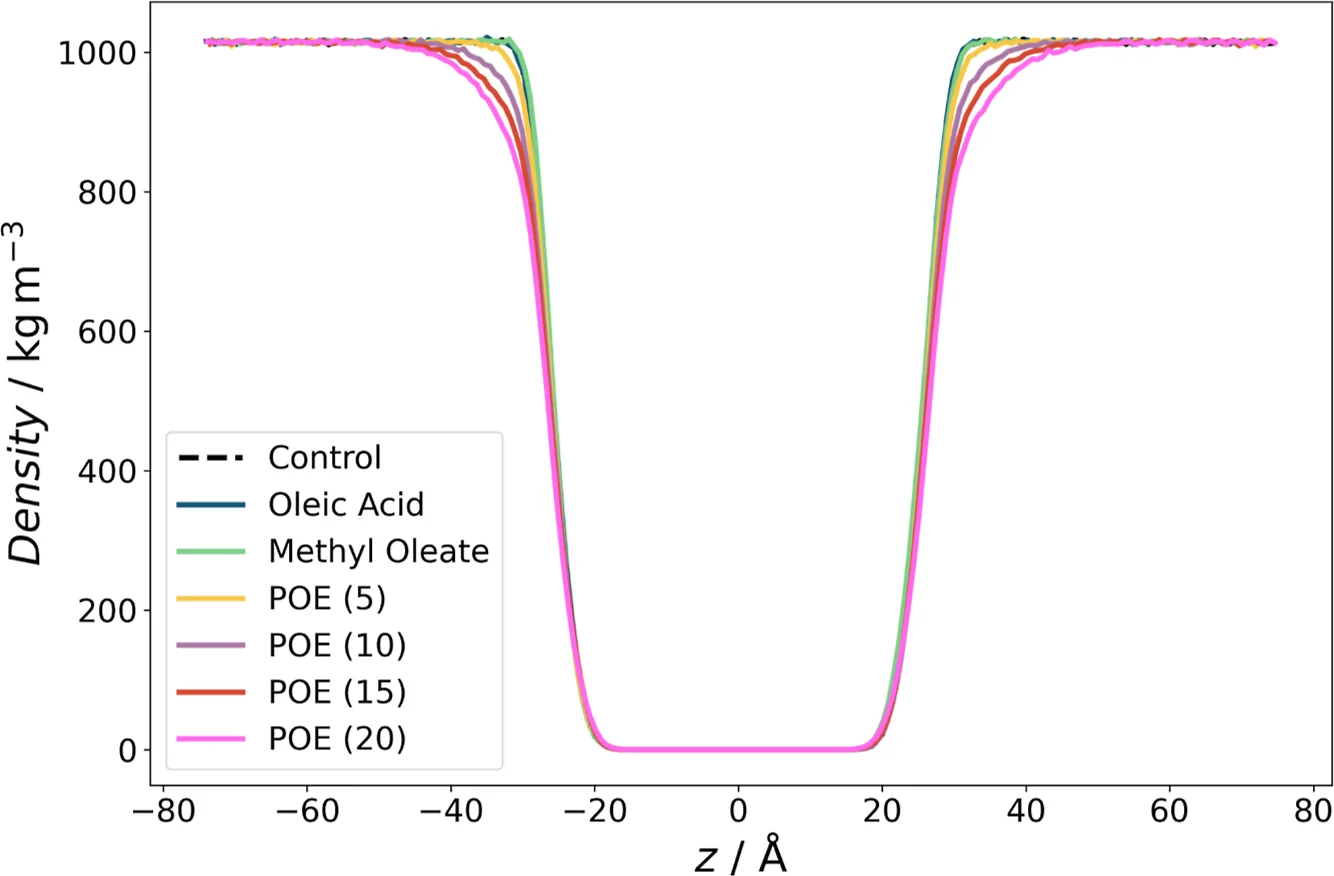
Water density profile for the control and oleyl homologue systems. Error not shown on profile, but found to be low across profile and below 7.5 kg m^–3^ at its highest point.

### Effects of Oleyl Homologue Inclusion on the Barrier to Water Permeation

To quantitatively determine the degree to which the oleyl homologues affect the barrier properties of the SC lipid bilayer, we used WPI to calculate the excess chemical potential profile of water across the bilayers. Figure 17 shows the resulting profiles for the control system and those containing each oleyl homologue. The addition of any of the oleyl homologues reduced the principal energy barrier to water permeation, which is located around 12.5 Å from the bilayer center. Oleic acid had the smallest overall effect, reducing the peak barrier height by 3 kJ mol^–1^ and slightly lowering the energy barrier in the bilayer core, corresponding to the dense lipid tail region. Methyl oleate had a similar effect on the peak barrier height but caused a more pronounced decrease in the bilayer core region around 0 Å. The inclusion of POE(5) and POE(10) oleyl ethers had only a small effect on the peak barrier heights but caused a slight narrowing of the overall chemical potential profile. Further lengthening of the POE headgroup to 15 and 20 units resulted in a greater decrease in both the peak barrier heights and the overall width of the profile. In summary, the addition of oleyl homologues decreased the barrier to water permeation in all cases, with POE(15) and POE(20) oleyl ethers having the most significant effect.

**Figure 17:**
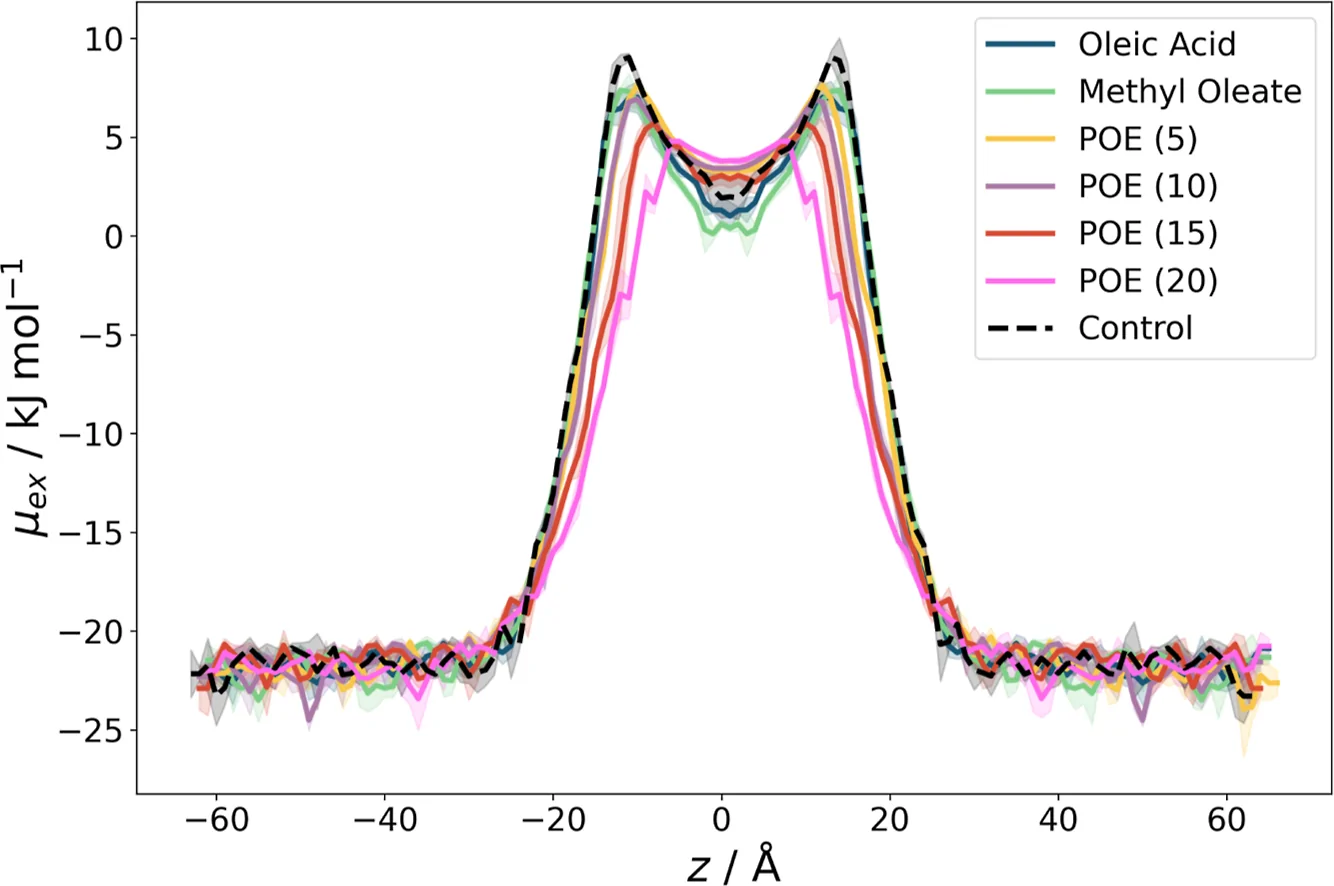
Excess chemical potential profile for water across control and oleyl homologue-containing SC lipid bilayer systems. Standard error is overlaid on each trace.

Convergence analysis is presented in Figure S8. Each of the three replicates for each system were observed to converge within the sampling time.

## Discussion

The purpose of this study was to use MD to investigate the skin penetration enhancing properties of a homologous series of oleyl-based coformulants and to explore underlying molecular mechanisms by which they modulate SC barrier function.

We first confirmed that all modeled oleyl homologues were capable of spontaneously inserting into SC lipid bilayers, where the polar carboxylic acid/methyl ester/POE headgroups associate with the SC lipid headgroups and the aliphatic oleyl tails associate with the other SC lipid tails. The time scales for partitioning varied considerably. Oleic acid and methyl oleate integrated fully within 100 ns, while POE oleyl ethers inserted more slowly and were not fully incorporated by 500 ns. This is likely due to increasing steric hindrance and reduced hydrophobicity as the POE chains increased in length. In all cases, membrane insertion was spontaneous, as expected for lipophilic compounds partitioning from an aqueous vehicle. Oleic acid has a very high octanol–water partition coefficient of 7.64, so it is unsurprising that partitioning occurs.^69^ Similarly, methyl oleate possesses a high octanol–water partition coefficient of 7.45, supporting our observation of spontaneous insertion.^70^ Exact octanol–water partition coefficient values for the POE oleyl ethers are not available.

To directly compare the effects of each oleyl homologue, we generated bilayers with 5 mol % of each oleyl homologue preinserted in the bilayer and assessed their impact on the bilayers. Across all systems, inclusion of oleyl homologues reduced the free energy barrier to water permeation, as evidenced by lower excess chemical potentials at the interface and near the bilayer core. This effect was most pronounced for POE(15) and POE(20). These observations are consistent with enhanced water permeability through the SC lipid bilayers. We note that this does not necessarily translate into increased permeability of active ingredients, which would likely vary by compound.

The trend in the chemical potential profiles correlated with decreased area compressibility moduli, indicating that all modeled oleyl homologues enhance the lateral deformability of the bilayer. This is likely driven by disruption of lipid packing and weakening of stabilizing interactions at the interface, including hydrogen bonding.

To understand the molecular basis for these results, we examined lipid order parameters. All oleyl homologues induced local disorder in the short tails of CER­[NP], particularly at carbons 2 and 3, which bear hydroxyl groups. The greatest effect was observed with the addition of oleic acid, likely due to its small, highly polar carboxylic acid headgroup being able to more effectively associate with and disrupt the hydroxyl bearing carbons.

Conversely, as we progress through the homologous series from oleic acid to methyl oleate and increasingly longer POE oleyl ethers, we observe a gradually increasing, but minor disruption of C_24_ fatty acid tail order. This trend is reflected in the local area per lipid: while oleic acid and methyl oleate induce minimal changes, POE oleyl ethers cause a gradual increase in the fatty acid area per lipid with increasing chain length.

Examining the bulk average area per lipid reveals little difference with oleyl homologue incorporation compared with the control system. However, oleic acid showed the lowest area per lipid of the oleyl homologues, with the substitution of the oleic acid with the methyl ester or POE ether resulting in a higher area per lipid for the oleyl homologue. We propose that this increase is due to the substitution of the polar carboxylic acid headgroup of oleic acid with less efficient hydrogen-bonding headgroups, reducing lipid packing efficiency.

Cholesterol packing was also consistently affected by oleyl homologue incorporation. Cholesterol molecules directly adjacent to the homologues showed a ∼10% increase in area per lipid along with a significant increase in the variance of the cholesterol tilt angle. Notably, this effect occurred across all the oleyl homologues, suggesting that the oleyl tail, i.e. the conserved structural motif, is primarily responsible for cholesterol disruption.

Hydrogen bond network analysis provided further insight into the destabilization of the bilayer. While oleic acid had little effect on the network, the inclusion of methyl oleate and POE oleyl ethers caused a gradual weakening of the hydrogen bond network, reflected by a systematic decrease in the lipid–lipid connections mediated through the extended hydrogen bonding network as well as the average transitivity and centrality. The weakening of the interfacial hydrogen bonding network correlates with a minor decrease in the order of the C_24_ fatty acids. These findings point to a steric blocking mechanism, where bulkier oleyl homologue headgroups impede water access to the interface and inhibit the formation of stabilizing hydrogen bonds between the lipids and interfacial solvent. This steric blocking also explains the sharp decrease in SASA observed with the inclusion of the POE oleyl ethers. Analysis of the bilayer surface showed that the location of solvent inaccessible regions coincided with the locations of POE headgroups, indicating that the flexible POE chains lie on the bilayer surface and physically block access to the underlying lipids.

Interactions between polar lipid headgroups and interfacial solvent are vital for maintaining lipid bilayer stability. The presence of long-chain POE ethers reduces the SASA, thereby limiting solvent interactions at the interface and effectively dehydrating the bilayer. This dehydration effect is also shown in the water density profile, where as the POE oleyl ether headgroup increases in length, the water density on the membrane surface decreases due to the steric blocking of the membrane interface. No additional water permeation into the membrane is observed, this is not expected over the simulated time scales, as there is still a moderate energy barrier to water permeation in all systems.

Overall, our findings show that oleyl homologues reduce the barrier function of the SC lipid matrix via multiple, cooperative mechanisms: (i) local disruption of CER­[NP] packing via headgroup interactions with hydroxyl-bearing carbons; (ii) minor local disordering of C_24_ fatty acid tails in the dense hydrocarbon core; (iii) disruption of cholesterol packing; and (iv) weakening of the hydrogen bonding network due to steric blocking, reduced headgroup polarity and lipid reorganization.

The results of our simulations align well with experimental findings on the effects of oleyl homologues on the SC skin barrier. Oleic acid is a widely recognized chemical penetration enhancer, known to increase the dermal absorption of a broad range of compounds.^18^ It is proposed that its action arises from fluidization of the SC lipids, primarily through interactions with and disruption of CER­[NS], and to a lesser extent, C_24_ fatty acids.^19,21^ Furthermore, oleic acid increases transepidermal water loss, consistent with a compromised barrier.^20^ Our simulations are in strong agreement with these observations, demonstrating increased water permeability in the presence of oleic acid as a result of decreased bilayer stability. Specifically, we observe disruption of SC ceramides via association with the hydroxyl-bearing carbons of the CER­[NP] short tail. In addition to this disruption, our simulations also reveal a minor loss of order in the C_24_ fatty acid chains and a disturbance in cholesterol packing.

Similar mechanisms are proposed for the dermal absorption enhancing effects of methyl oleate, chiefly lipid fluidization.^26^ We show from our simulations that methyl oleate shows a decrease in the free energy barrier of water permeation by inducing the same disorder as oleic acid, with an additional disruption to the interfacial hydrogen bond network.

POE fatty acid ethers are shown to significantly enhance transepidermal water loss by both disrupting lipid ordering and extracting lipids, especially for POE ethers of length 20.^29,30^ This is well reflected in the chemical potential profiles, with all modeled POE ethers showing decreases in the water permeability barrier, and POE(20) showing the greatest decrease of all the modeled oleyl homologues. Although we do not see lipid extraction on our simulation time scales, as we lengthen the POE lipid headgroup, we see the increasing interference of the interfacial hydrogen bonding network vital to sustaining the SC membrane structural organization.^58^

It should be noted that the effects of oleyl homologues observed in our simulations are likely to be concentration dependent; however, a systematic study of such concentration-dependent behavior lies beyond the scope of the present study. Furthermore, in realistic formulations, additional physical processes, such as competition between incorporation into bulk solvent aggregates and partitioning into the SC lipid matrix, will influence the effective oleyl homologue concentration, but are not captured in these simulations. Therefore, using a fixed 5 mol % concentration enabled direct comparison of the effects of different homologues on membrane properties and permeability.

It is widely observed in experiments that oleic acid and propylene glycol have a synergistic effect in enhancing dermal absorption by greater SC disruption.^71,72^ Experiments with the pharmaceutical tenoxicam show oleic acid and propylene glycol have a synergistic effect in enhancing skin permeability, with formulations containing both oleic acid and propylene glycol demonstrating higher dermal absorption values than either on their own.^73^ Work by Mistry and Notman suggests that propylene glycol enhances skin permeation by associating with the SC lipid headgroups and impeding the formation of hydrogen bonds with interfacial water molecules, inducing disorder within the SC lipid bilayer.^74^ Our work seems to suggest that the POE oleyl ethers have a mechanism of action similar to the synergistic effects of both oleic acid and propylene glycol, in a single molecule.

The SC model utilized in this study represents a simplified, three-component idealized lipid environment for computational feasibility. Our model approximates the short periodicity phase of the SC, omitting the more complex long periodicity phase. Additionally, to model the incorporation of cosolvents, we simulated a fully hydrated lipid bilayer, which does not fully capture the low-hydration, multilamellar structure characteristic of the SC lipid matrix. Furthermore, we employ a bilayer configuration in which ceramides typically adopt a hairpin conformation; native SC ceramides often adopt an extended conformation.^75^ These simple models provide a foundational view of the SC lipid interactions, although we acknowledge they may influence the direct translation of the results to the true behavior of the human SC.

## Conclusion

In this study, we have carried out simulations of model SC lipid bilayers containing 5 mol % inclusions of a series of oleyl homologues. All the modeled oleyl homologues were found to reduce the free energy barrier to water permeation. This effect was most pronounced for the POE oleyl ethers, with longer POE chains having the greatest effect. Mechanistically, we show that these oleyl homologues disrupt SC lipid organization via several cooperative effects. All tested homologues locally disrupt cholesterol packing. The short tails of CER­[NP] were also disrupted, particularly at the hydroxyl-bearing carbons, due to interactions with the oleyl homologue headgroups. In the case of the POE oleyl ethers, increasing headgroup length led to progressive blocking of the bilayer surface, which weakened the interfacial hydrogen bonding network and slightly reduced the C_24_ fatty acid tail order. Future work aims to explore more potential SC coformulant interactions, as well as the role of these effects in more complex solvent/formulation environments.

To our knowledge, this is the first MD study to investigate the interactions of methyl oleate and POE oleyl ethers with skin lipids. These findings provide valuable molecular-level insights into how these common coformulants modulate skin barrier function and should inform the rational design and safety assessment of dermal formulations.

## Supplementary Material

















## Notes

OLEP.txt: Oleic acid gromacs topology file (TXT)
MOLEP.txt: Methyl oleate gromacs topology file (TXT)
OPGV.txt: POE(5) gromacs.itp topology file (TXT)
OPGX.txt: POE(10) gromacs.itp topology file (TXT)
OPGXV.txt: POE­(15) gromacs.itp topology file (TXT)
OPGXX.txt: POE(20) gromacs.itp topology file (TXT)
SI.pdf: Supporting Information file (PDF)
tpi.txt: Patched gromacs tpi.cpp file (TXT)
