# Supplementary material for: Skin Barrier Modulation by Oleyl-Based Co-Formulants: Mechanistic Insights from Molecular Simulations
Source: ACS Omega. 2026 Aug 5;11(32):47424–39. doi: 10.1021/acsomega.6c00967 (PMC13491466; doi:10.1021/acsomega.6c00967)
Supplement: Supplementary file 1 [file ao6c00967_si_007.pdf]

Skin Barrier Modulation by Oleyl-Based Co-Formulants: Mechanistic Insights from Molecular Simulations - Supporting Information

Callum J.W.R. Ward,[*,†] Conor Whitehouse,[‡] Jennifer Webb,[‡] and Rebecca Notman[*,¶]

†*Department of Chemistry, University of Warwick, Coventry CV4 7AL, United Kingdom*

‡*Jealott's Hill International Research Centre, Syngenta, Bracknell RG42 6EY, United Kingdom*

¶*School of Biochemistry, University of Bristol, Bristol BS8 1TD, United Kingdom*

E-mail: *callum.ward@warwick.ac.uk; *rebecca.notman@bristol.ac.uk

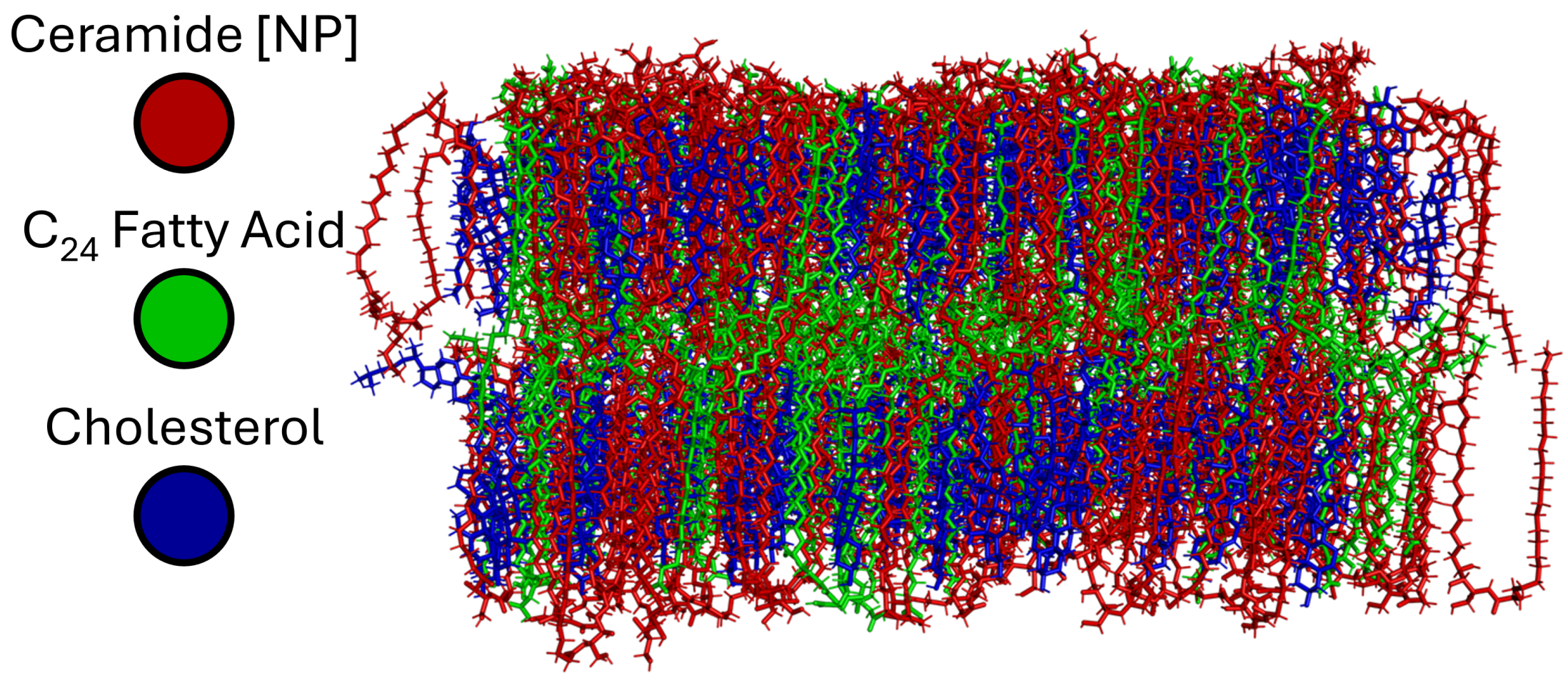

Figure S1: Snapshot of modelled SC lipid membrane.

1 Methods

1.1 Area Per Lipid

We use Voronoi tessellation to calculate the area per lipid and generate an accurate spatial map of our lipid leaflets for localised analysis. For each lipid we select a range of headgroup atoms to define the headgroup spatial extent. For cholesterol, we select the oxygen of the hydroxyl, for the other lipids, a single atom does not accurately capture the headgroup spatial extent, so we select three atoms to fully describe the headgroup. These assignments are available in Figure S2.

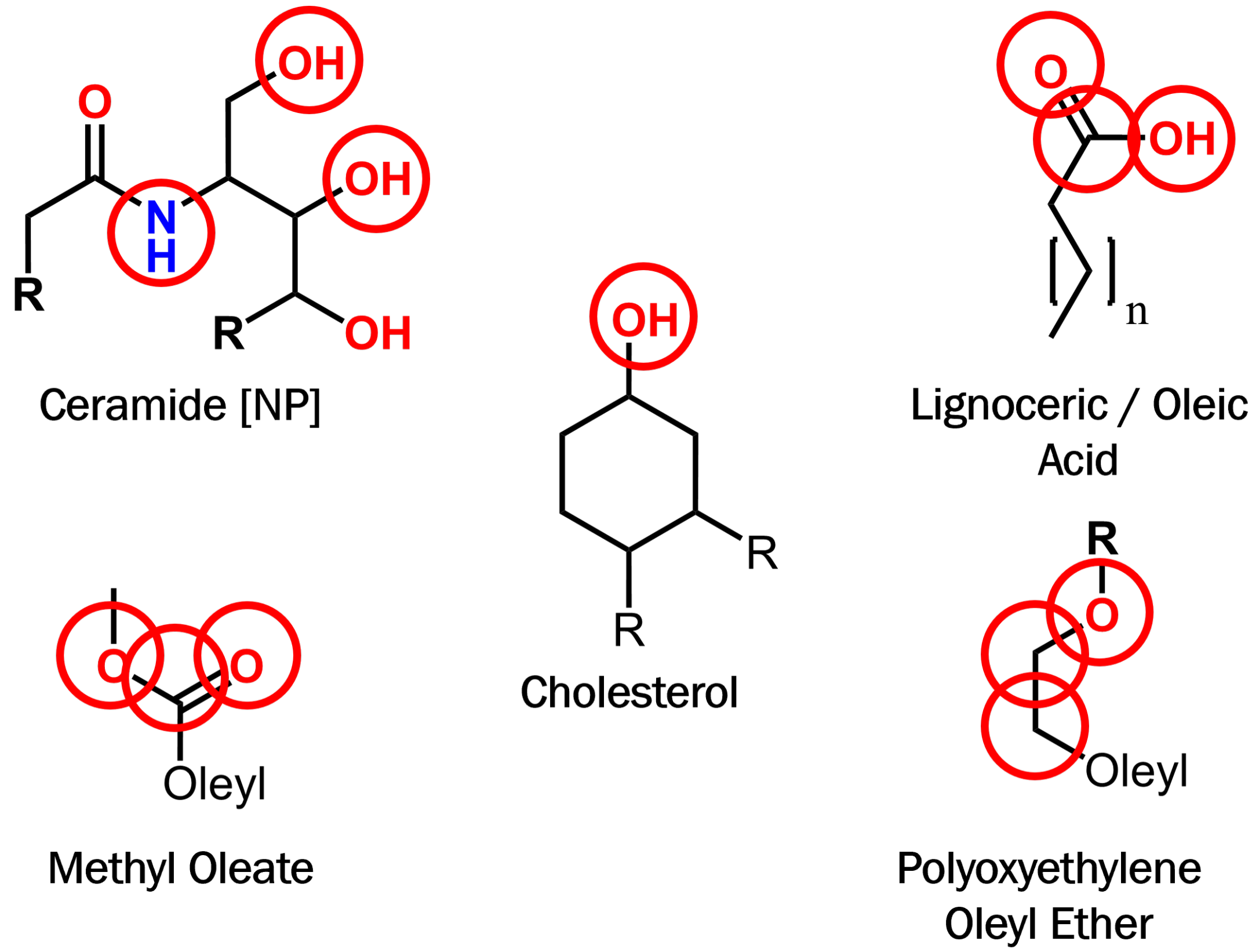

Figure S2: The headgroup atoms used for Voronoi analysis are circled in red for ceramide, C_{24} fatty acid, oleyl homologues and cholesterol

1.2 Widom Particle Insertion

Before applying Widom particle insertion to our skin-lipid systems, we decided to validate the method on a dipalmitoylphosphatidylcholine (DPPC) bilayer and compare with previous Widom particle insertion calculations performed by *Saito* and *Shinoda*[1]. The CHARMM GUI bilayer builder was used to generate an initial bilayer structure containing 159 DPPC lipids per leaflet and a 5 nm thick water padding on each side of the membrane giving 30,535 water molecules. Simulations were performed using the GROMACS 2023.3 simulation package.[2–4] The all-atom CHARMM36 force field[5] and TIP3P water model were used.[6]

Simulations of this DPPC bilayer were performed at 323.15 K and 1 atm, using the v-rescale thermostat and c-rescale barostat, with coupling constants of 1 ps and 5 ps, respectively. Pressure was applied semi-isotropically along the *xy* and *z* axes. Solvent and bilayer

atoms were coupled separately in the thermostat. Non-bonded interactions were treated using a cutoff scheme for short-range interactions and particle-mesh Ewald (PME) for long-range electrostatics.[7] A cutoff of 1.2 nm was applied to both van der Waals and short-range electrostatic interactions. For van der Waals interactions, a force-switching function was applied between 1.0 and 1.2 nm. Covalent bonds involving hydrogen atoms were constrained using the LINCS algorithm.[8] Periodic boundary conditions were applied in all three spatial dimensions.

Equilibration run parameter files were provided by CHARMM GUI. Systems were first energy-minimised using the steepest descent algorithm, followed by two NVT equilibration steps of 125 ps, each with a 1 fs timestep, and with decreasing *xyz* position restraints from 1000 to 400 $\mathrm{kJ\,mol^{-1}\,nm^{-2}}$. These were followed by a series of four NPT equilibration steps of 250 ps each with a 2 fs timestep, and with position restraints gradually reduced from 400 to 0 $\mathrm{kJ\,mol^{-1}\,nm^{-2}}$. Following this, production simulations were run for 150 ns using a 2 fs timestep.

Test particle insertions were performed on the full 150 ns of the production simulation, totalling 15,000 frames. Within each frame, 15,000 random insertions were attempted inside a sampled 75 Å×75 Å×2 Å slab in the center of the *xy* plane. Each insertion was repeated 50 times, where the water molecule was randomly rotated and displaced (no more than 0.25 Å) about its initial test position. Sampling was performed on slabs 1 Å apart, with a slab thickness of 2 Å, from 1.5 nm to 12 nm along the z axis, resulting in 105 test particle insertion simulations along the z axis.

Widom particle insertion was performed, both with and without long-range electrostatic interactions. Short range interaction energies were calculated using a cutoff of 1.2 nm applied to both van der Waals and short-range electrostatic interactions. For van der Waals interactions, a force-switching function was applied between 1.0 and 1.2 nm, and a simple shifted cutoff was applied to the short-range electrostatics. Additionally, we tested a reaction field electrostatic potential with a dielectric constant of ∞ and real space cutoff of 1.2 nm

to simply model a long-range diffuse electrostatic interaction. When considered, explicit long-range electrostatics were modelled using the PME method with a real-space cutoff of 1.2 nm.

We found that including both short and long-range electrostatics produces very different excess chemical potential profiles. When only considering short-range electrostatics, a far larger energy barrier is predicted compared with the profile calculated using PME. *Saito* and *Shinoda* calculate a peak barrier of $\sim$5.0 kcal mol^{-1}, comparing well with our PME system. Including a reaction field potential improves upon the simple shifted cutoff scheme but still does not reproduce the previously modelled free energy barrier. These verification simulations revealed that performing Widom particle insertion without explicit consideration of long-range electrostatics results in the overestimation of the excess chemical potential barrier. On the basis of these results we proceeded to use PME for the treatment of electrostatics in the Widom particle insertion calculations presented in this paper.

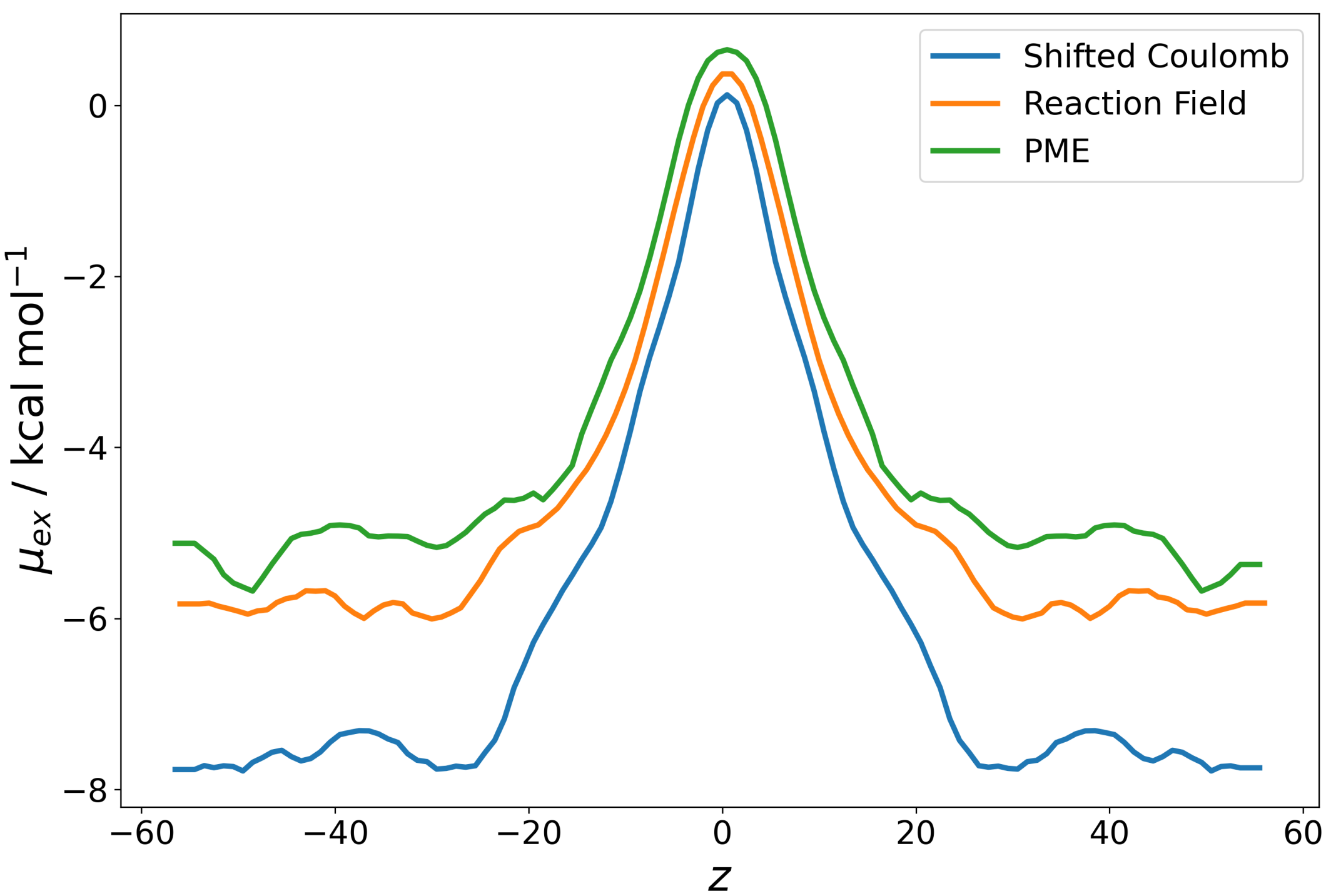

Figure S3: Comparison of excess chemical potential calculated using Widom particle insertion with shifted coulomb potentials, reaction field and PME methods. PME was shown to closely match previous calculations by Saito and Shinoda.

1.3 Hydrogen Bond Network Transitivity

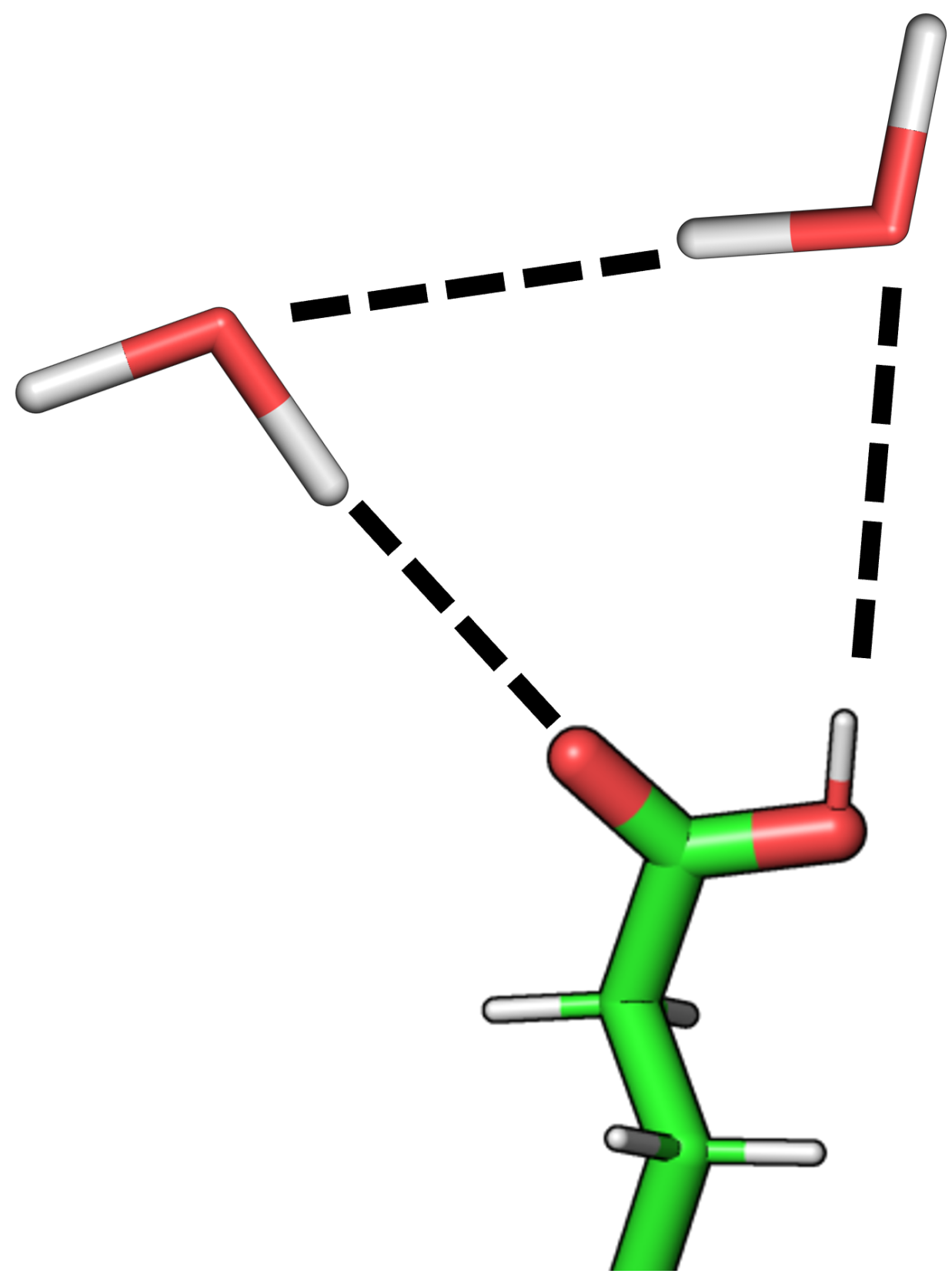

Figure S4: Example of a closed triplet hydrogen bond cluster that would contribute to the transitivity of the hydrogen bond network.

2 Results

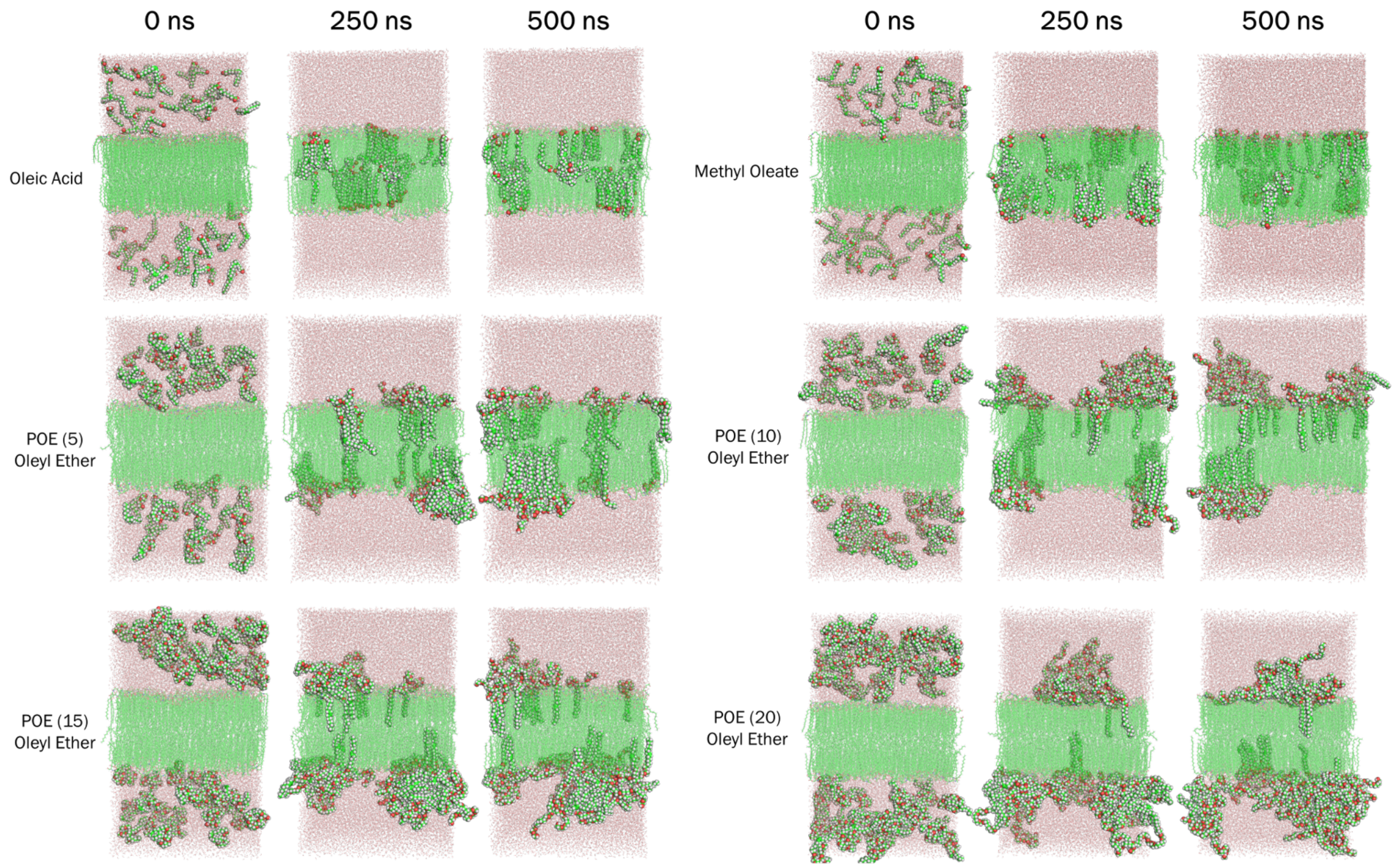

Figure S5: Snapshots from oleyl homologue spontaneous insertion simulations between 0 and 500 ns

Table ST1: Comparison of the average number of hydrogen bonds formed between SC membrane constituents and interfacial solvent molecules in each simulated system.

	Control	Oleic Acid	Methyl Oleate	POE(5) Oleyl Ether	POE(10) Oleyl Ether	POE(15) Oleyl Ether	POE(20) Oleyl Ether
**CER[NP]-CER[NP]**	227.30 ± 1.40	229.79 ± 1.30	221.88 ± 2.80	225.58 ± 1.20	224.88 ± 1.35	235.80 ± 0.74	232.53 ± 0.87
**CER[NP]-C_{24} Fatty Acid**	64.01 ± 1.26	67.59 ± 0.61	72.61 ± 2.51	76.41 ± 0.91	76.18 ± 0.45	72.91 ± 0.69	81.91 ± 1.16
**CER[NP]-Cholesterol**	126.69 ± 2.51	113.89 ± 1.58	119.87 ± 2.36	122.30 ± 0.42	129.24 ± 2.11	113.79 ± 2.37	114.93 ± 1.01
**C_{24} Fatty Acid - C_{24} Fatty Acid**	3.44 ± 1.75	3.24 ± 2.12	2.70 ± 4.47	2.59 ± 1.66	2.91 ± 2.75	3.13 ± 0.62	2.89 ± 1.17
**C_{24} Fatty Acid - Cholesterol**	10.07 ± 0.10	10.74 ± 0.05	9.84 ± 0.03	10.67 ± 0.04	11.61 ± 0.07	12.56 ± 0.07	12.29 ± 0.07
**Cholesterol - Cholesterol**	1.46 ± 0.18	0.21 ± 0.30	0.11 ± 0.21	0.22 ± 0.22	0.00 ± 0.23	0.00 ± 0.17	0.00 ± 0.20
**CER[NP]-Water**	692.72 ± 1.40	697.42 ± 0.39	701.29 ± 2.87	683.10 ± 0.72	665.04 ± 1.62	637.72 ± 1.82	633.54 ± 1.48
**C_{24} Fatty Acid - Water**	330.97 ± 0.68	323.92 ± 0.07	314.85 ± 0.05	304.32 ± 0.10	296.11 ± 0.00	301.28 ± 0.00	281.04 ± 0.00
**Cholesterol - Water**	276.10 ± 1.03	284.45 ± 0.83	280.27 ± 0.96	272.05 ± 1.27	263.66 ± 2.66	274.17 ± 2.21	272.14 ± 1.63

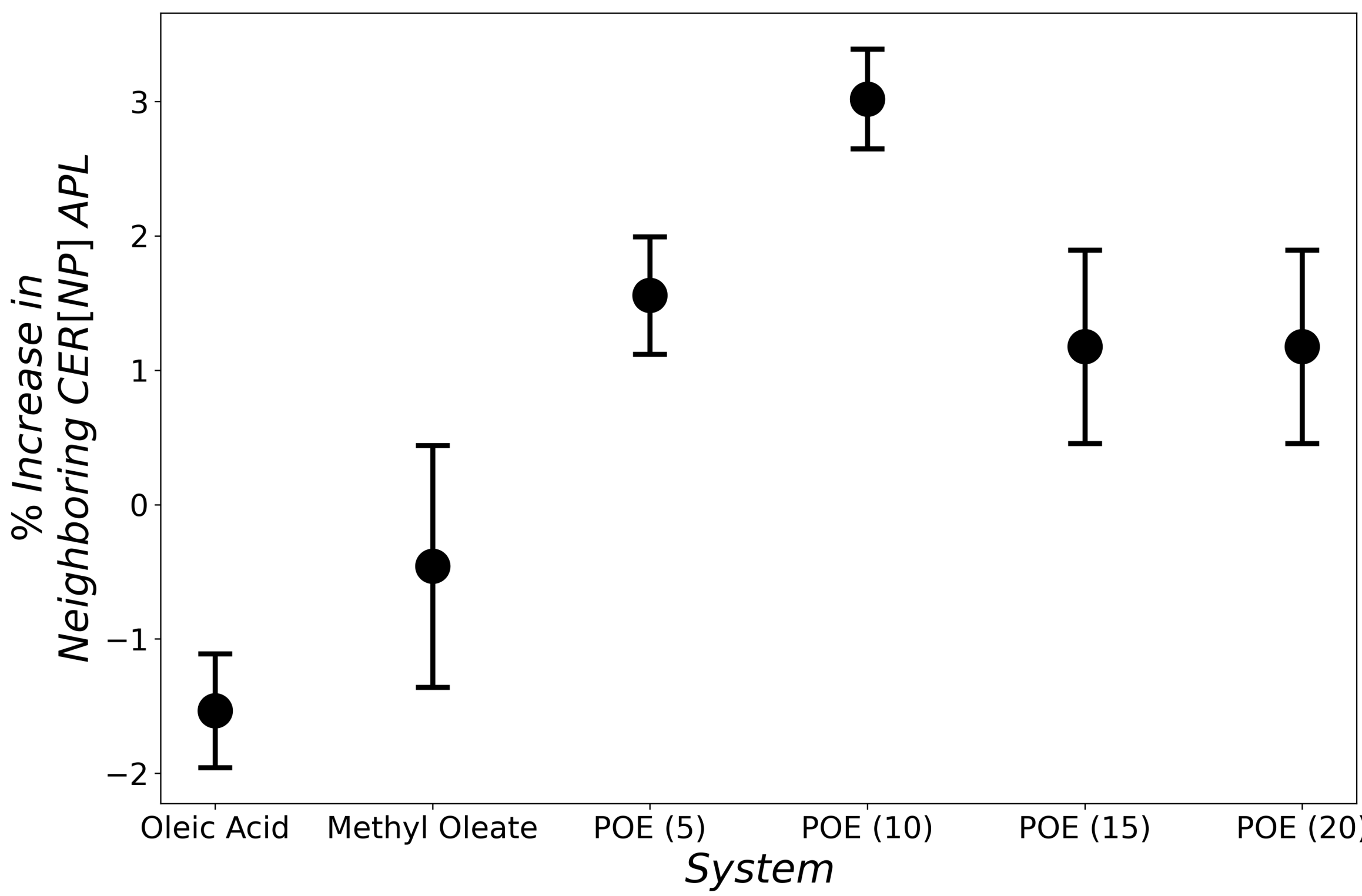

Figure S6: Average increase in area per lipid of CER[NP] molecules directly adjacent to oleyl homologues compared to the bulk average CER[NP] area per lipid.

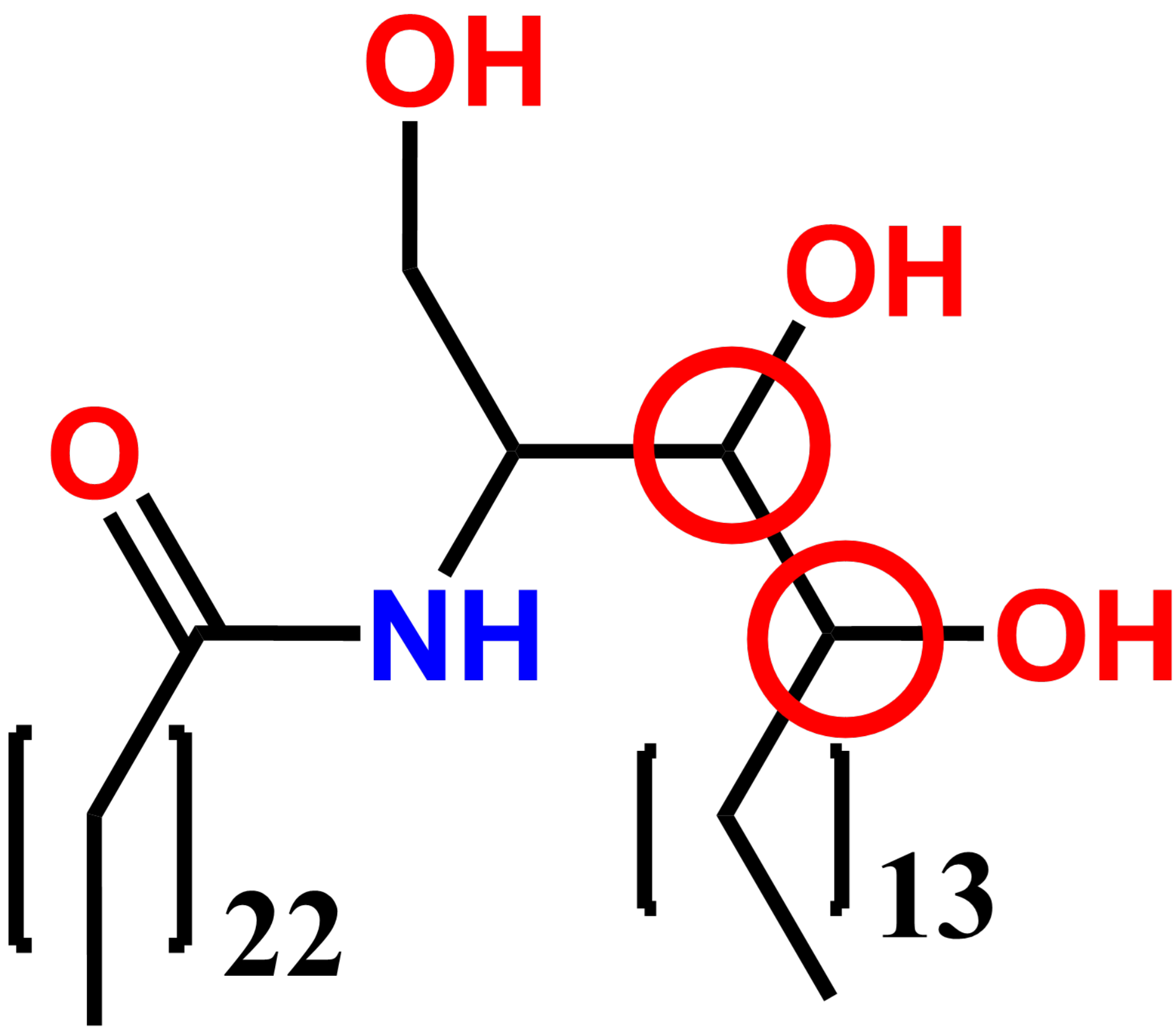

Figure S7: CER[NP] headgroup highlighting hydroxyl-bearing carbons (C2 and C3) implicated in local tail disordering.

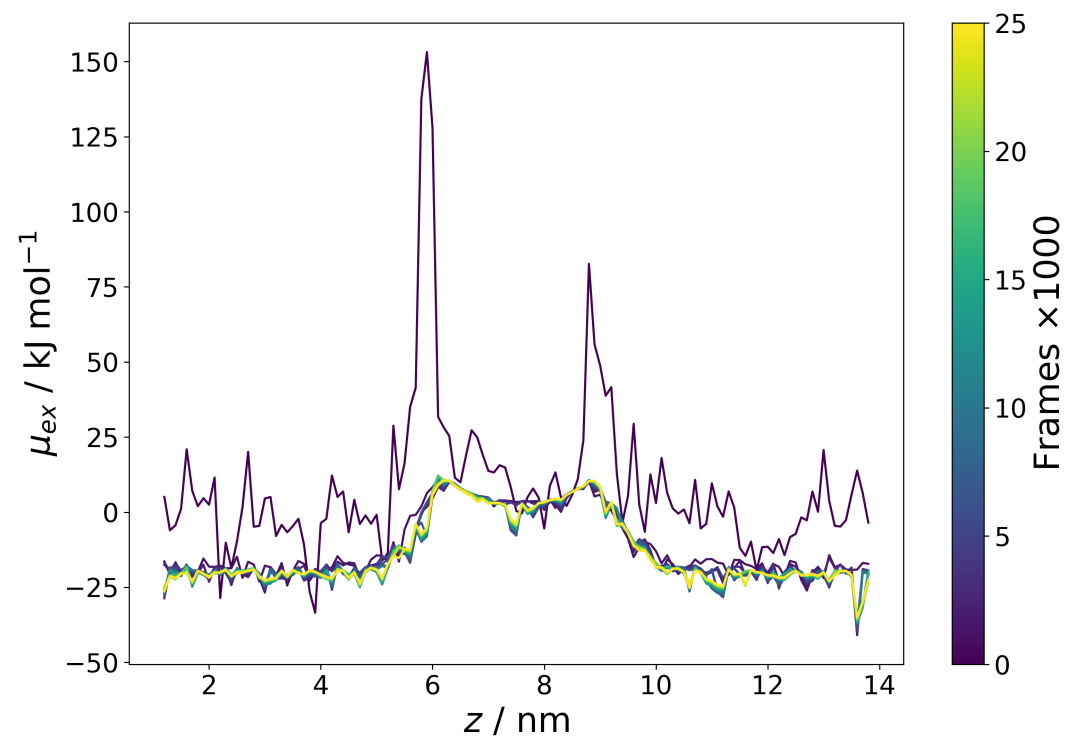

Control

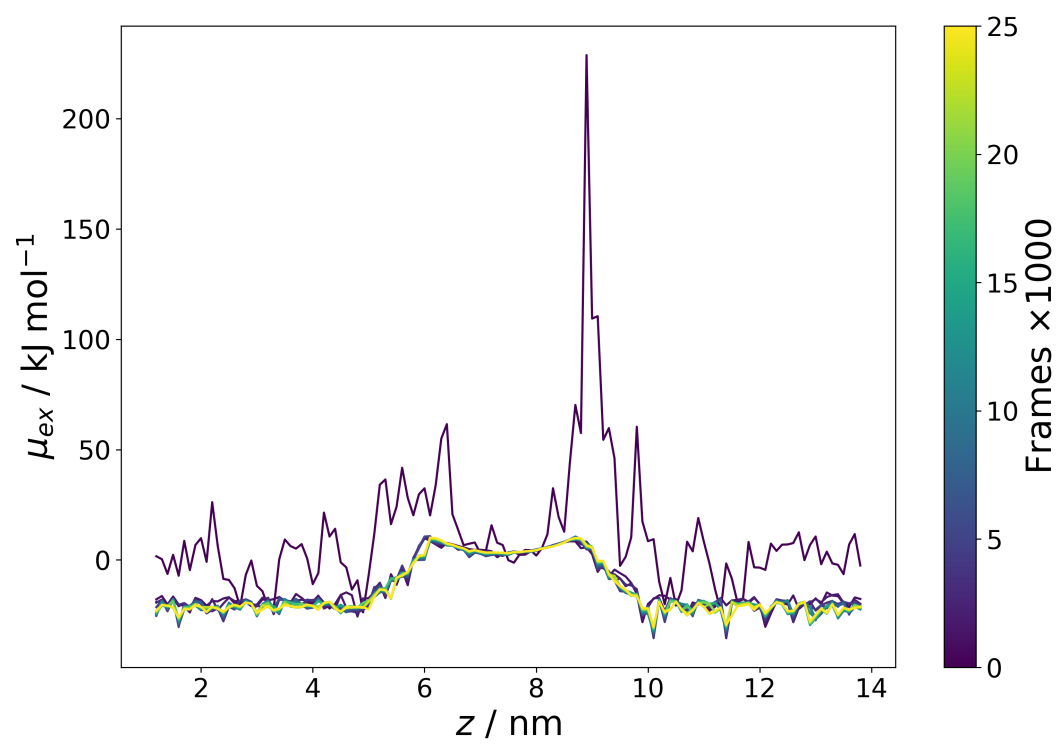

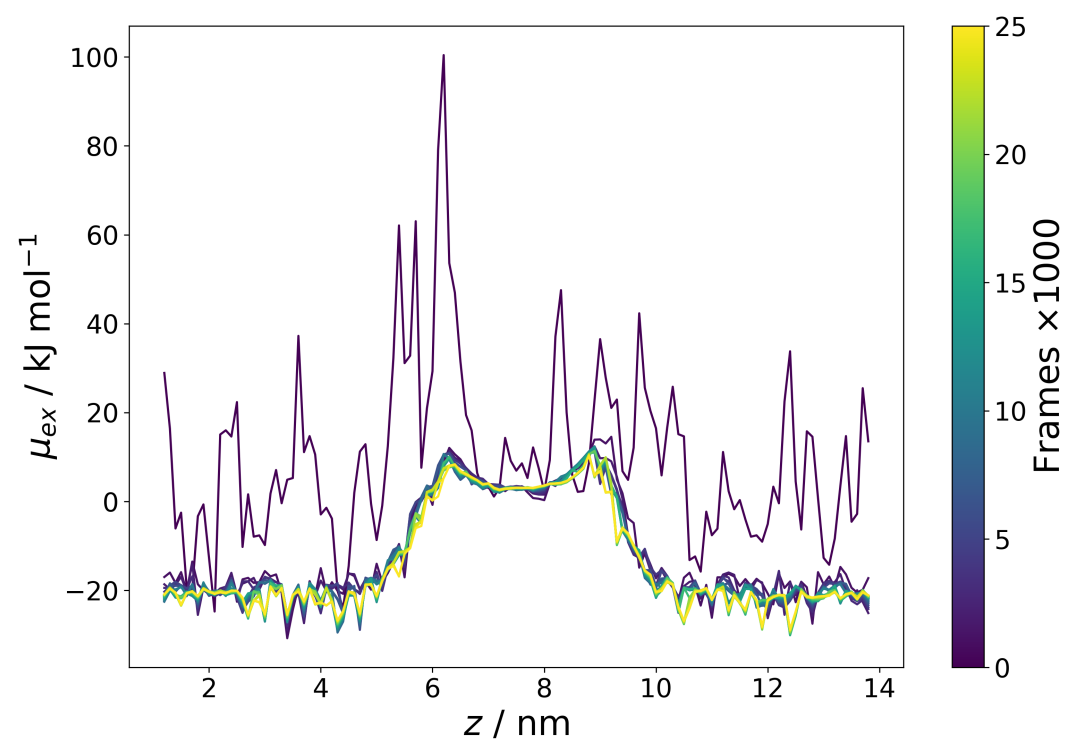

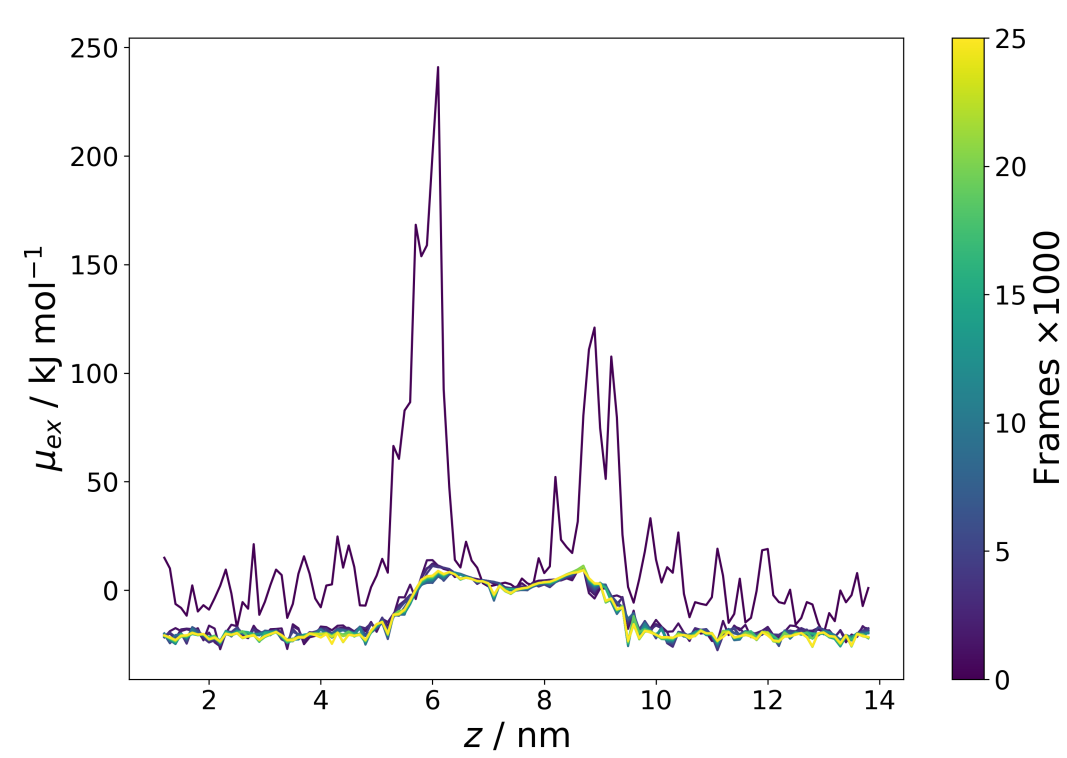

Oleic Acid

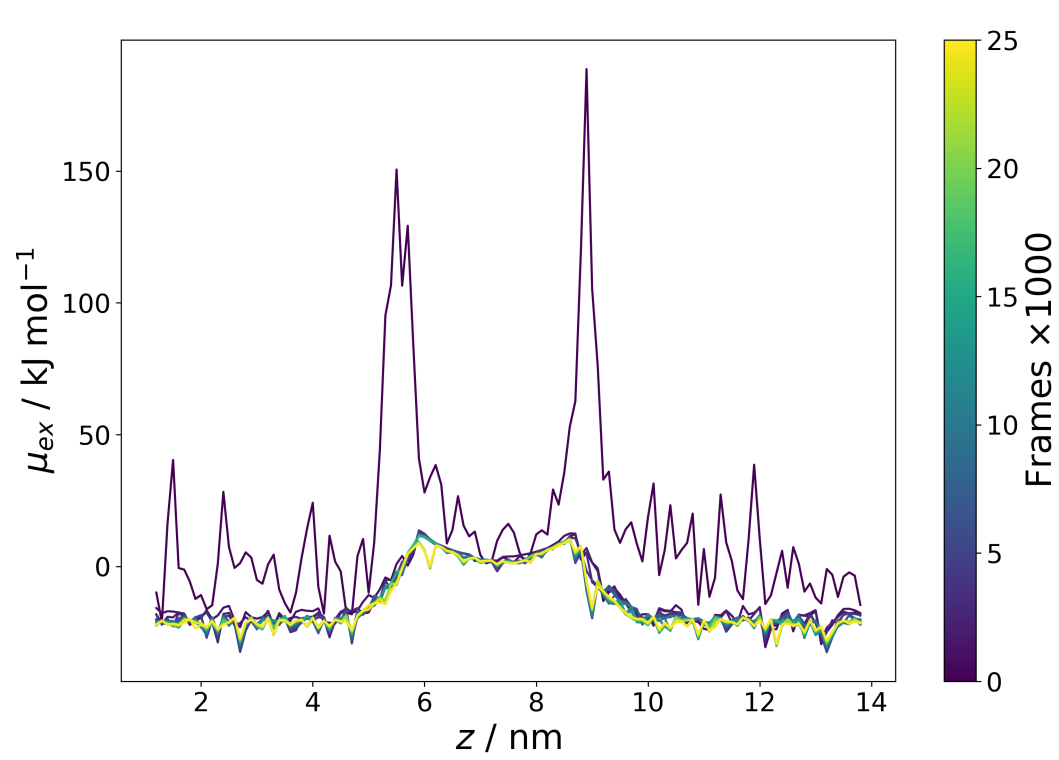

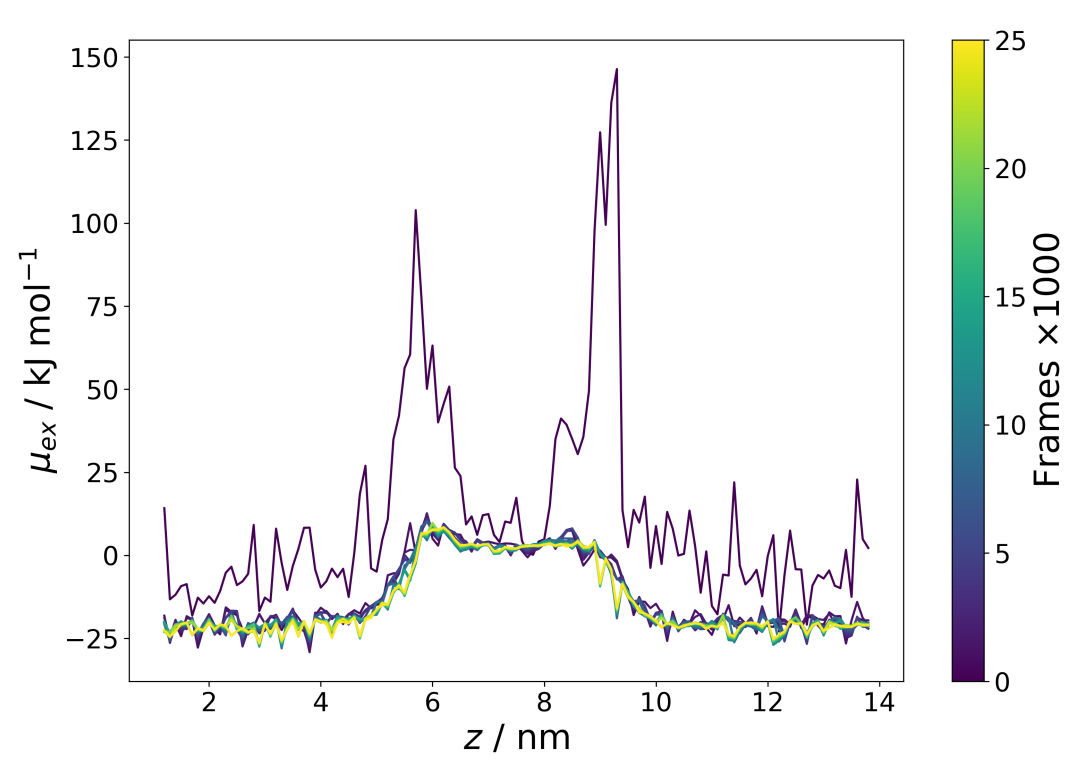

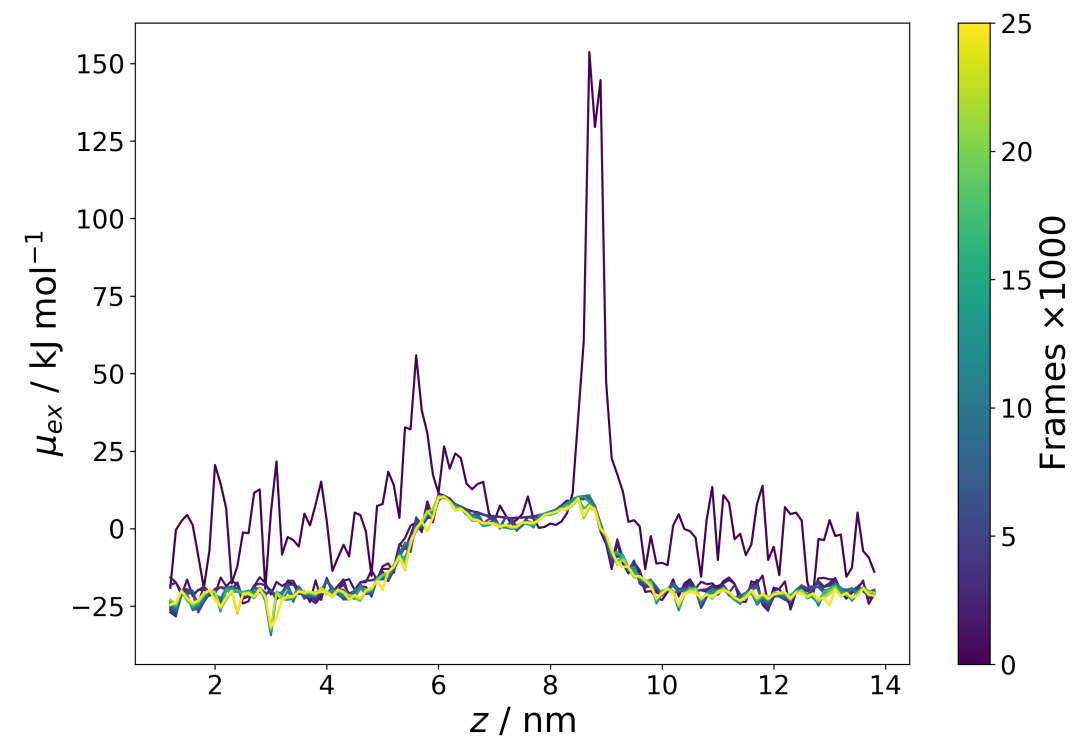

Methyl Oleate

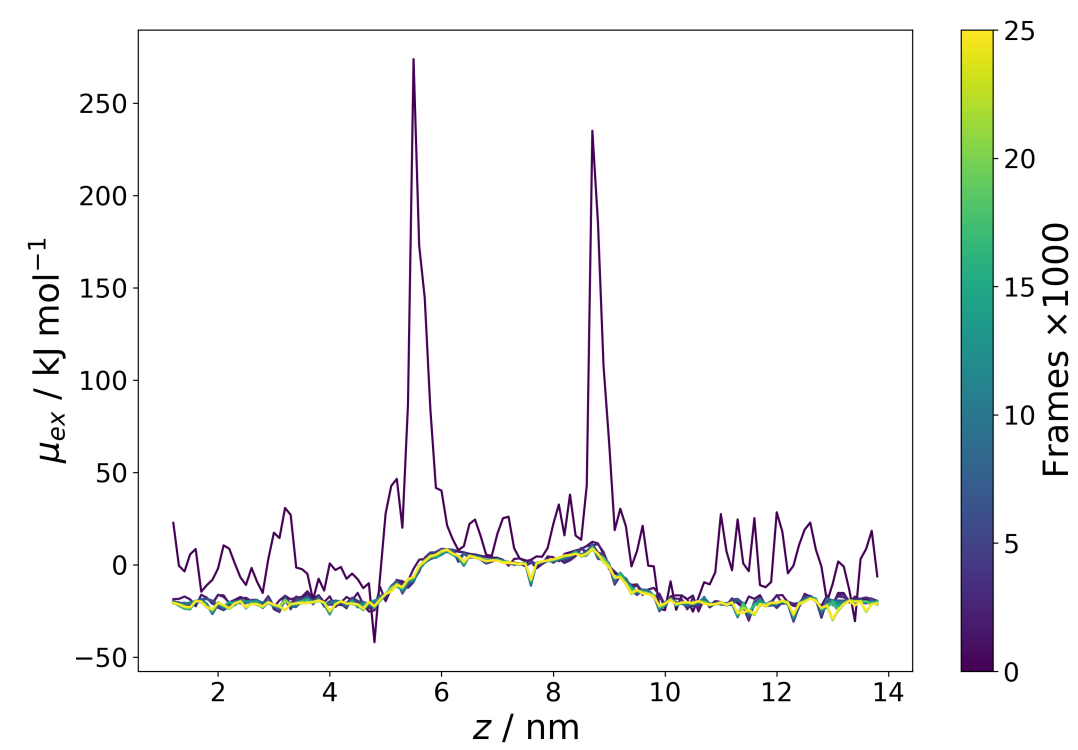

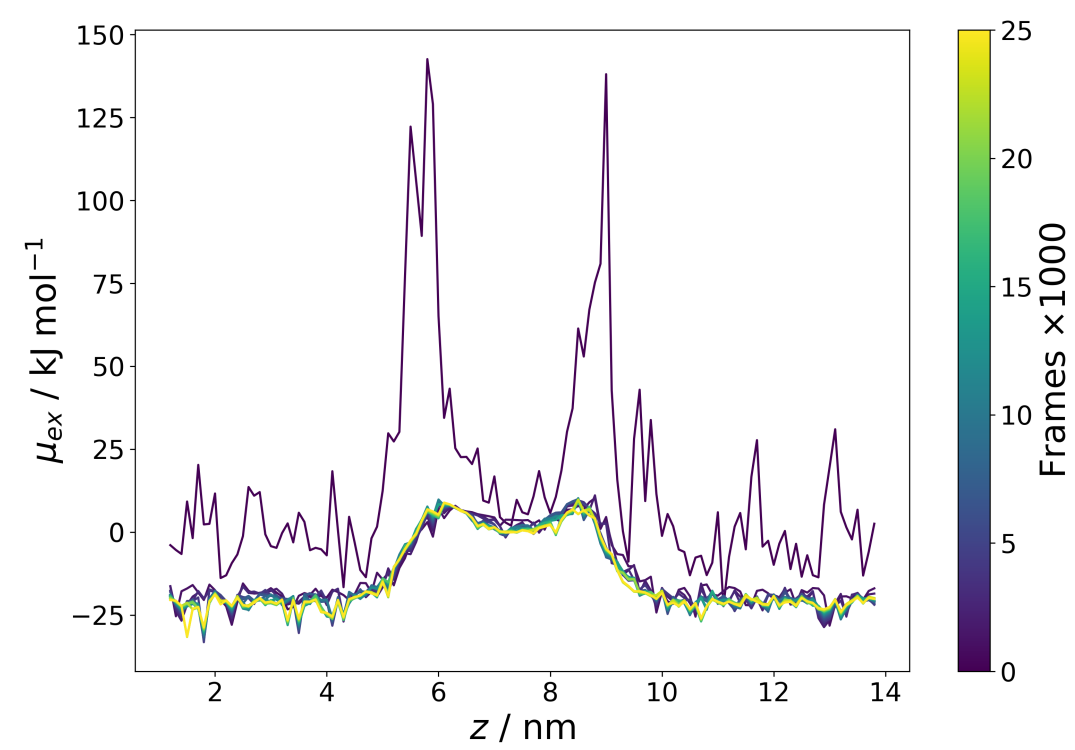

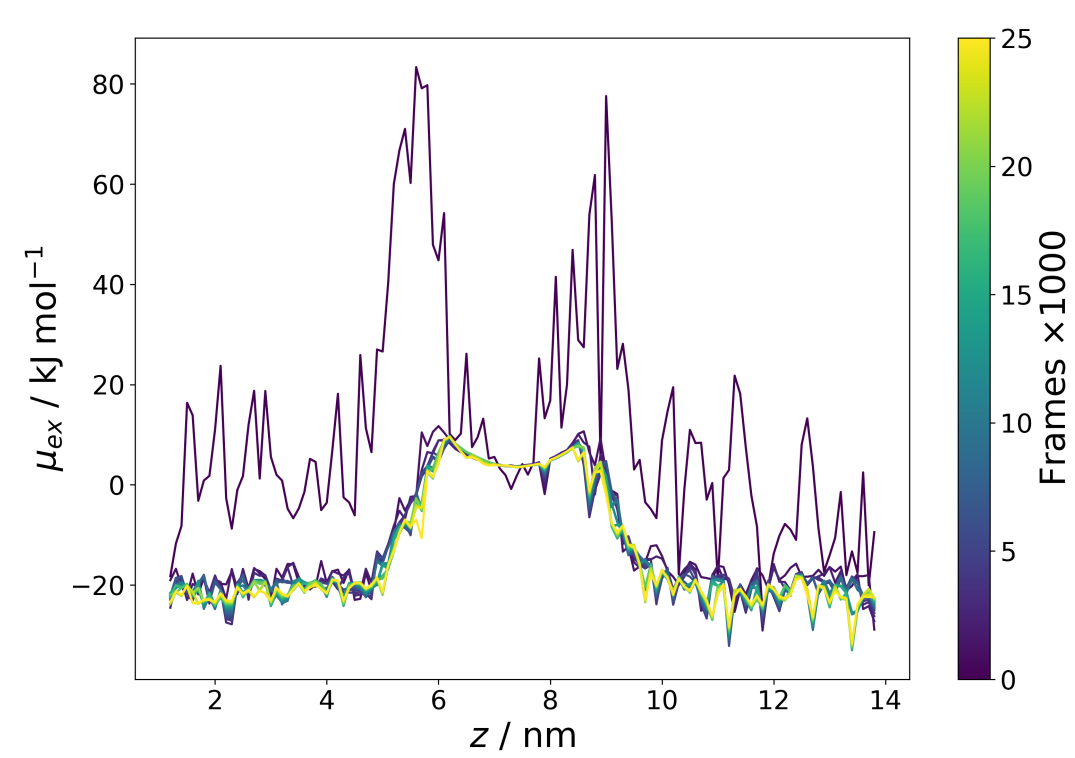

POE(5) Oleyl Ether

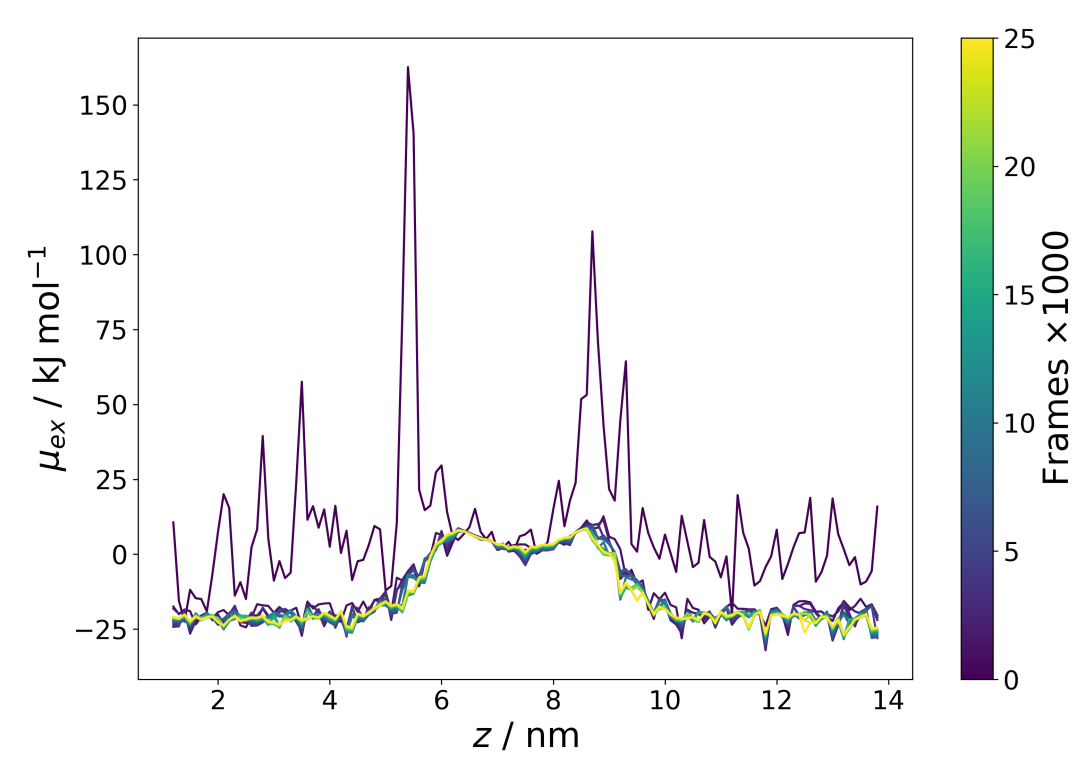

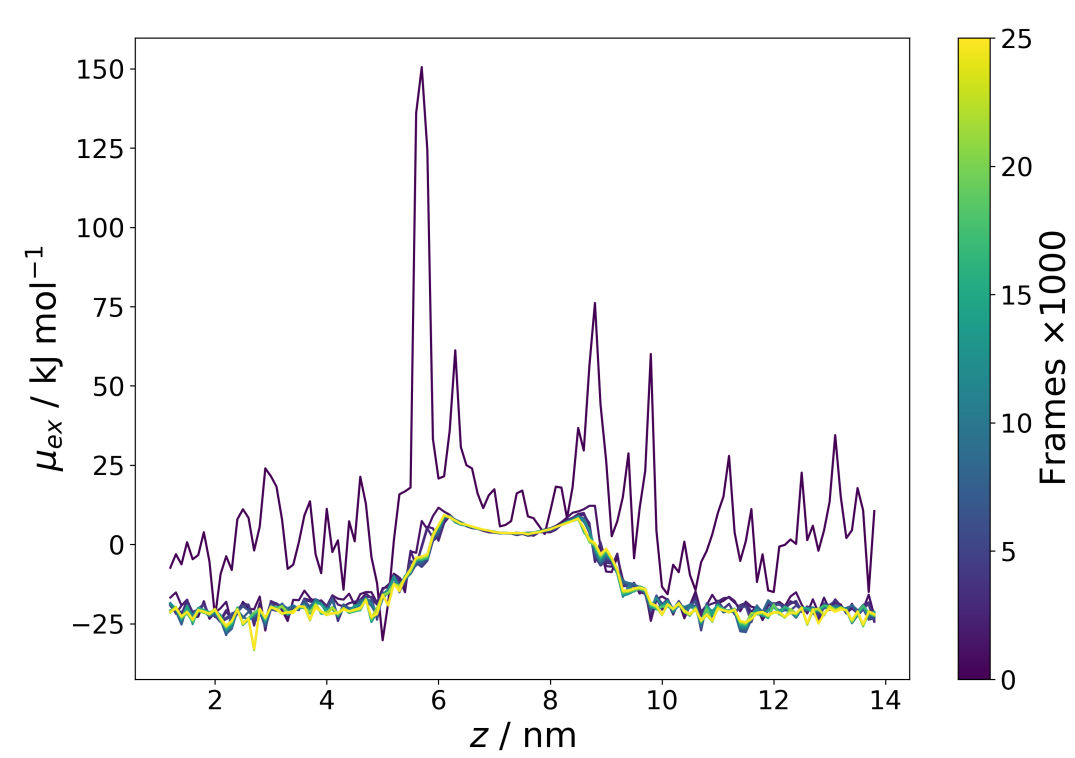

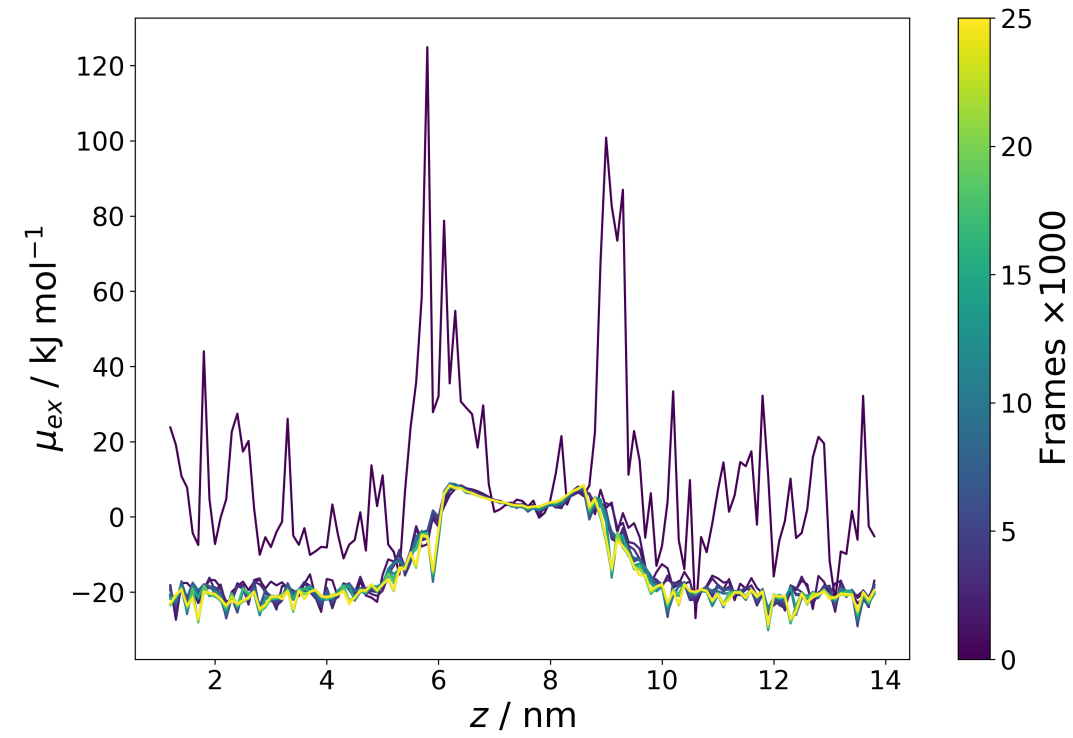

POE(10) Oleyl Ether

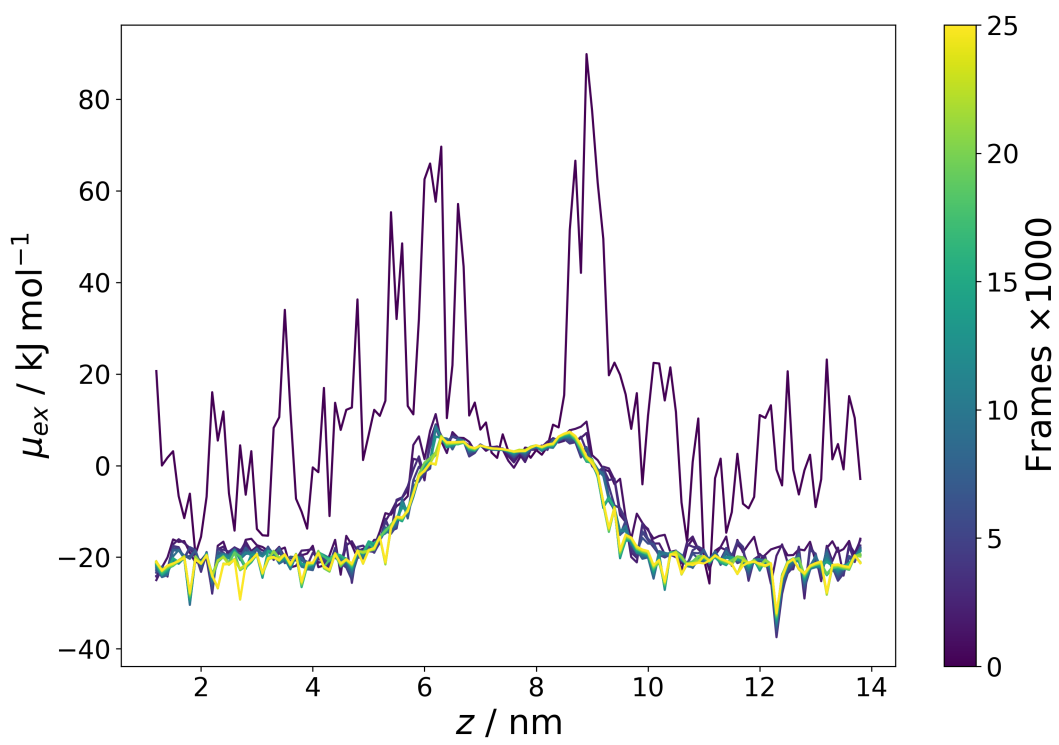

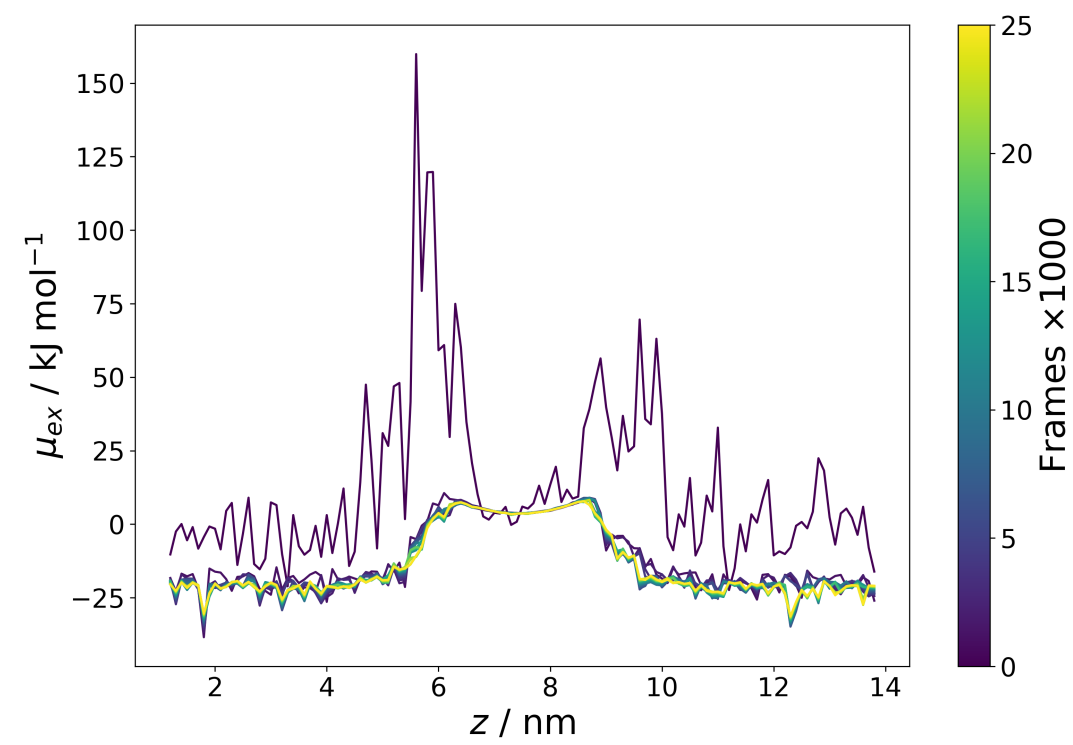

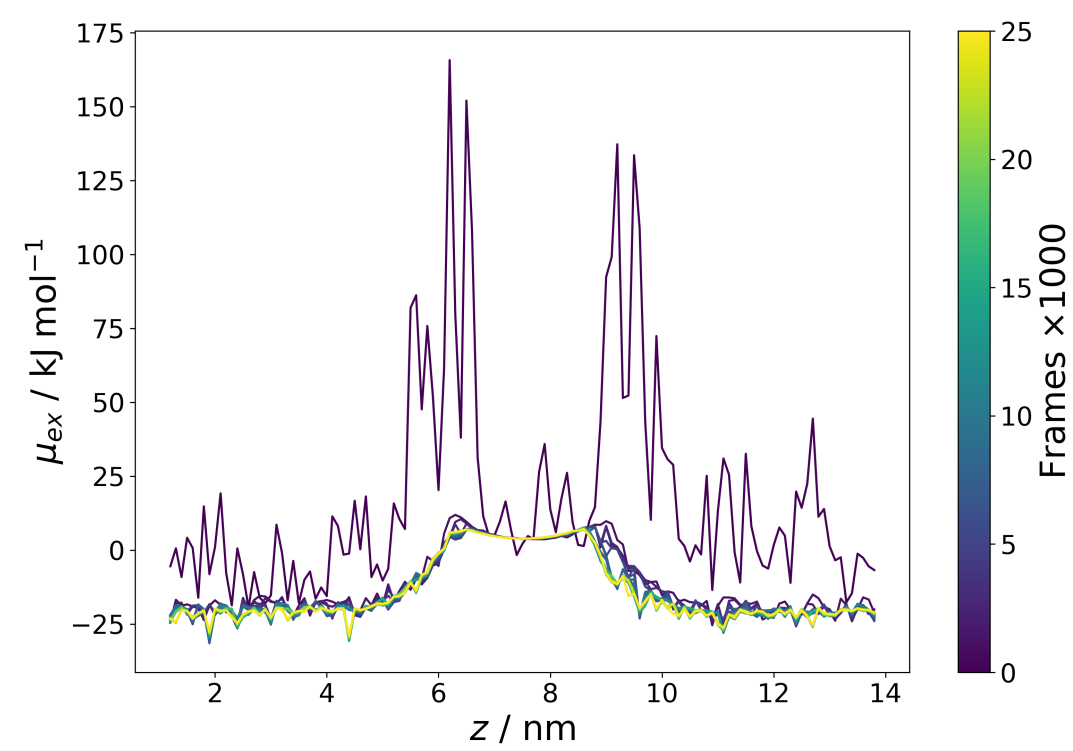

POE(15) Oleyl Ether

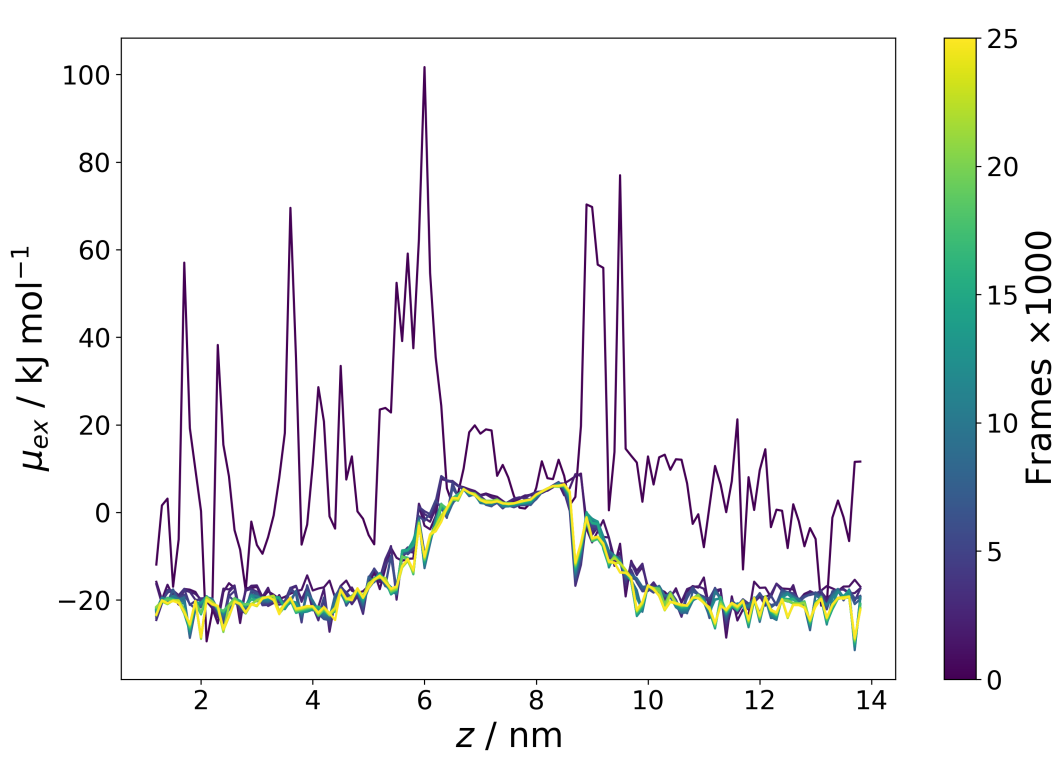

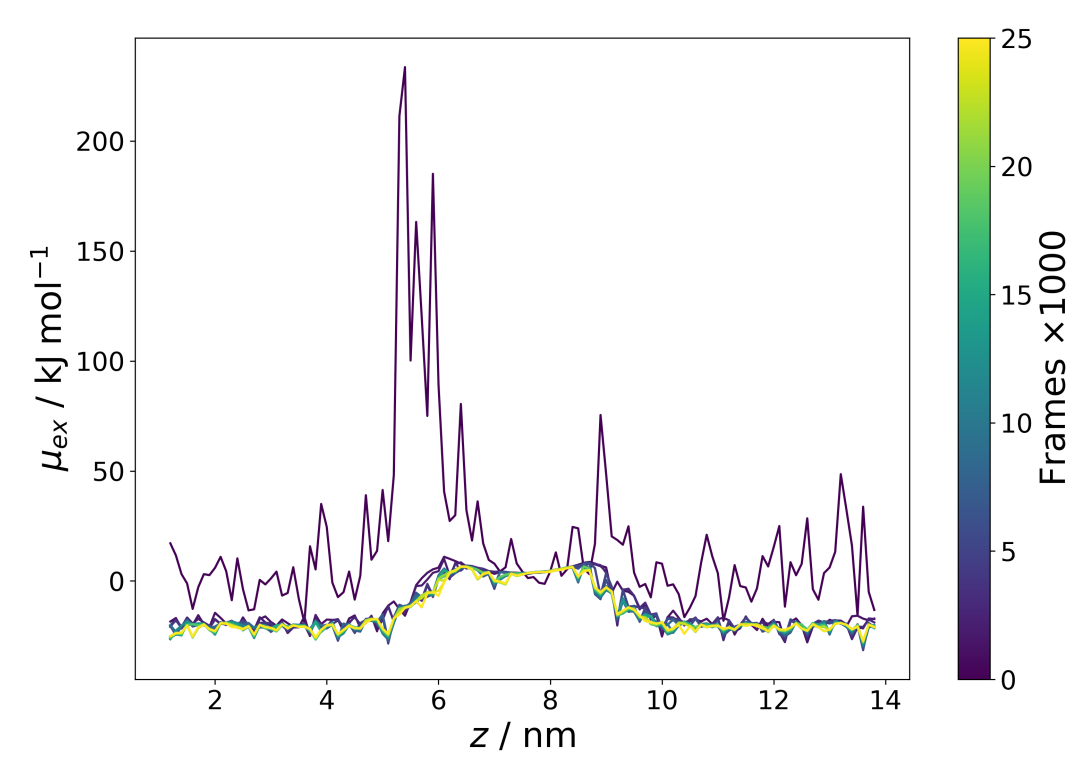

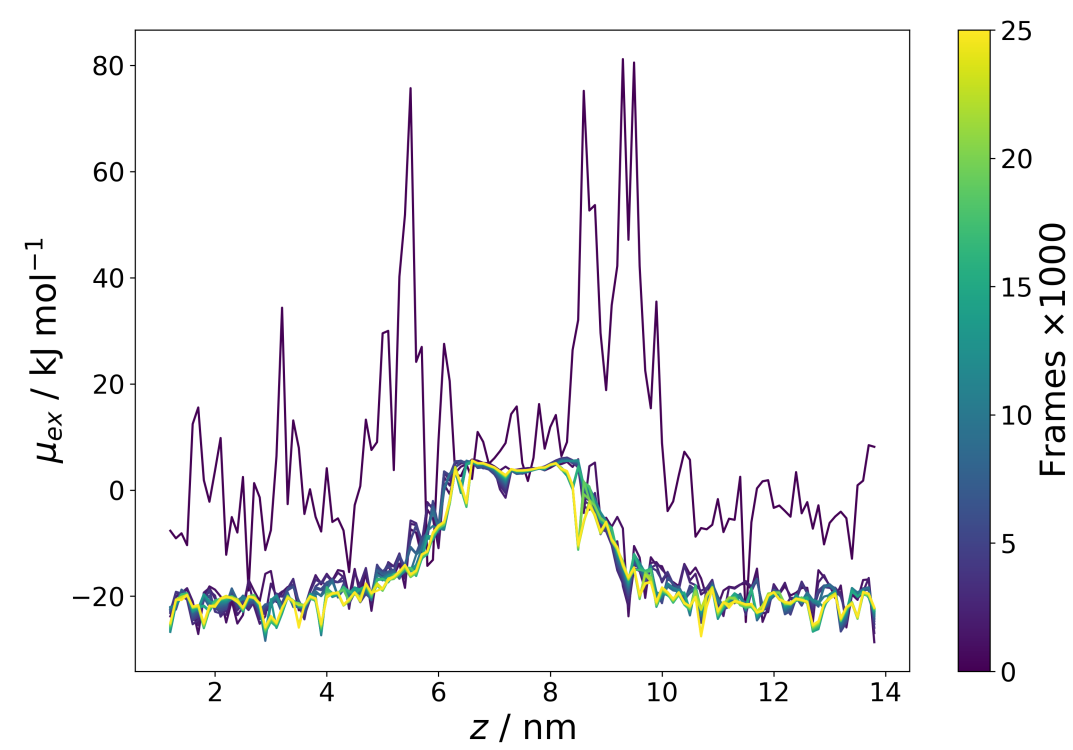

POE(20) Oleyl Ether

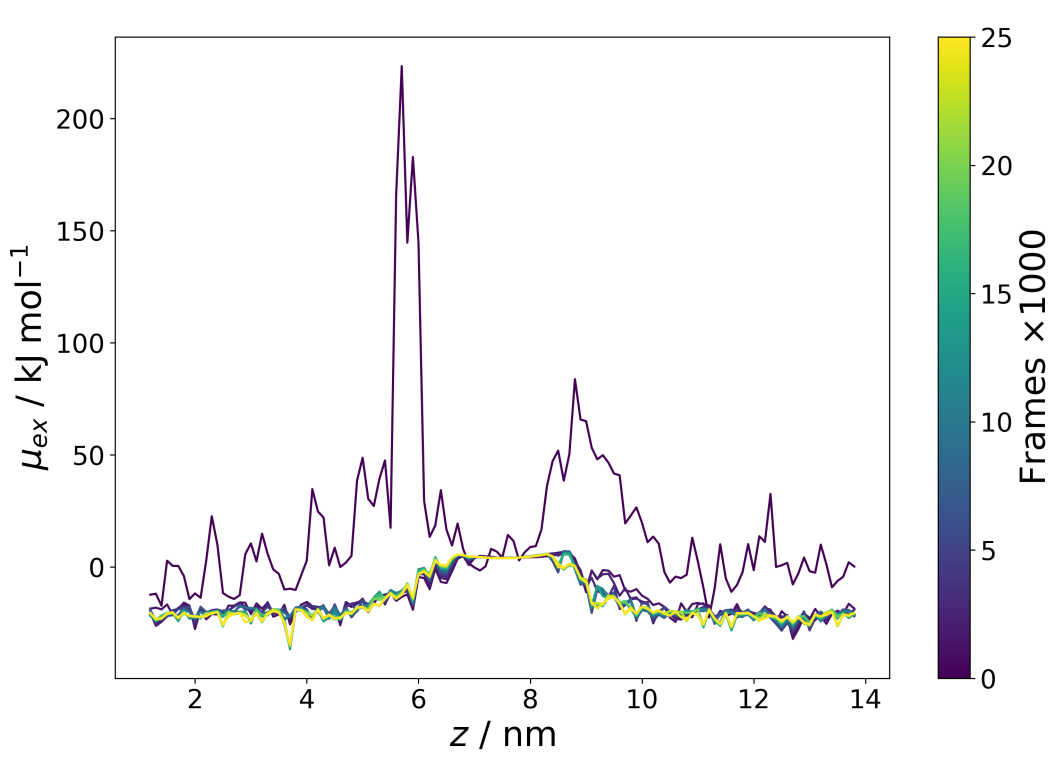

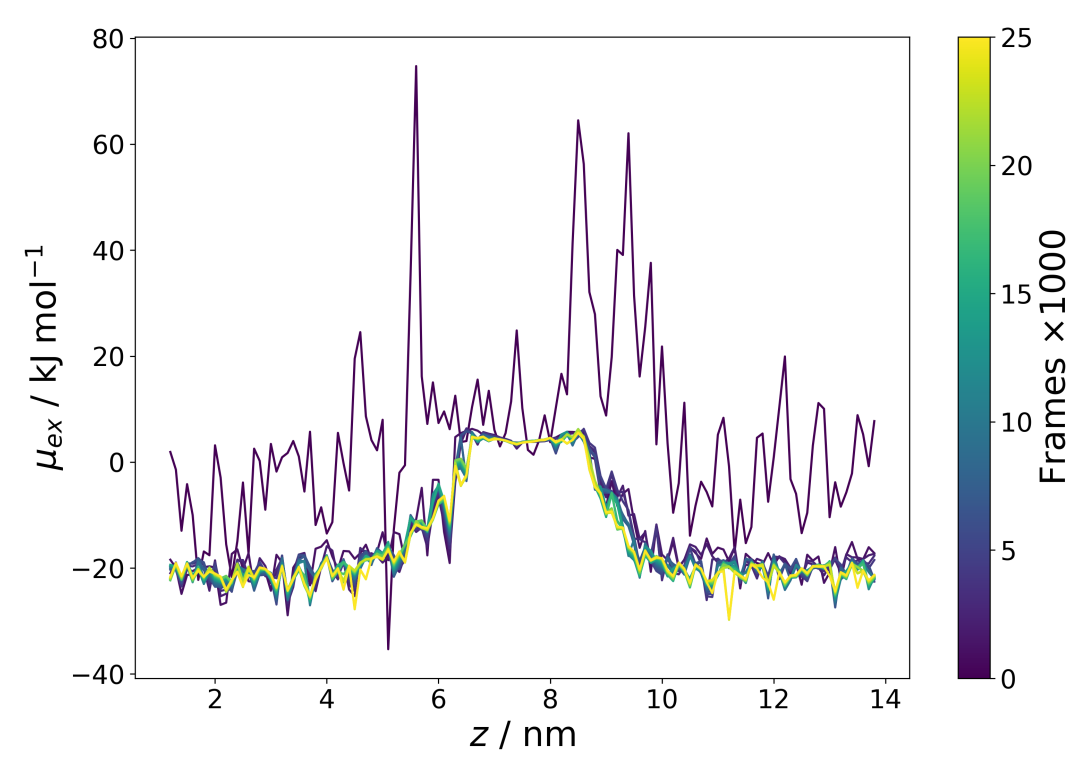

Figure S8: Convergence of Widom particle insertion calculations for each of the three replicates for each system, sampled every 1000 simulation frames showing convergence with time.

References

(1) Saito, H.; Shinoda, W. Cholesterol Effect on Water Permeability through DPPC and PSM Lipid Bilayers: A Molecular Dynamics Study. *The Journal of Physical Chemistry B* **2011**, *115*, 15241–15250.

(2) Bekker, H.; Berendsen, H.; Dijkstra, E.; Achterop, S.; Drunen, R.; van der Spoel, D.; Sijbers, A.; Keegstra, H.; Reitsma, B.; Renardus, M. GROMACS: A parallel computer for molecular dynamics simulations. *Physics Computing* **1993**, *92*, 252–256.

(3) Berendsen, H.; van der Spoel, D.; van Drunen, R. GROMACS: A message-passing parallel molecular dynamics implementation. *Computer Physics Communications* **1995**, *91*, 43–56.

(4) Abraham, M. J.; Murtola, T.; Schulz, R.; Páll, S.; Smith, J. C.; Hess, B.; Lindahl, E. GROMACS: High performance molecular simulations through multi-level parallelism from laptops to supercomputers. *SoftwareX* **2015**, *1-2*, 19–25.

(5) Best, R. B.; Zhu, X.; Shim, J.; Lopes, P. E. M.; Mittal, J.; Feig, M.; MacKerell, A. D. J. Optimization of the Additive CHARMM All-Atom Protein Force Field Targeting Improved Sampling of the Backbone , and Side-Chain 1 and 2 Dihedral Angles. *Journal of Chemical Theory and Computation* **2012**, *8*, 3257–3273.

(6) Jorgensen, W. L.; Chandrasekhar, J.; Madura, J. D.; Impey, R. W.; Klein, M. L. Comparison of simple potential functions for simulating liquid water. *The Journal of Chemical Physics* **1983**, *79*, 926–935.

(7) Darden, T.; York, D.; Pedersen, L. Particle mesh Ewald: An Nlog(N) method for Ewald sums in large systems. *The Journal of Chemical Physics* **1993**, *98*, 10089–10092.

(8) Hess, B.; Bekker, H.; Berendsen, H. J. C.; Fraaije, J. G. E. M. LINCS: A linear constraint solver for molecular simulations. *Journal of Computational Chemistry* **1997**, *18*, 1463–1472.
